# Supplementary material for: Complete functional analysis of type IV pilus components of a reemergent plant pathogen reveals neofunctionalization of paralog genes
Source: PLoS Pathog. 2023 Feb 13;19(2):e1011154. doi: 10.1371/journal.ppat.1011154 (PMC9956873; doi:10.1371/journal.ppat.1011154)
Supplement: S1 Table — (PDF) [file ppat.1011154.s002.pdf]

Table S1. List of *pil* and associated genes deleted in *X. fastidiosa* strain TemeculaL.

Locus ID[a]	Gene	Genome annotation[a]	General description	Reference
**Major pilin[b]**				
PD1924	*pilA1*	Type IV fimbrial precursor	Major pilin subunit that composes the main structural component of TFP	1
PD1926	*pilA2*	Fimbrial protein		
PD1077	*pilA3*	Fimbrillin		
**Minor pilin**				
PD0024	*pilE1*	Type IV pilin	Minor pilins responsible for priming the assembly of TFP and promoting the display of the PilY1 adhesin at the tip of the pilus	2
PD1610	*pilE2*	Type IV pilin		
PD0020	*pilV1*	Prepilin leader sequence		
PD1614	*pilV2*	Type IV pilus modification protein		
PD0021	*pilW1*	Prepilin type N-terminal cleavage//methylation domain		
PD1613	*pilW2*	Pilus assembly protein		
PD0022	*pilX1*	Type IV fimbrial biogenesis protein		
PD1612	*pilX2*	Type IV fimbrial biogenesis protein		
PD0019	*fimT1*	Prepilin like leader sequence	Minor pilin that interacts directly with PilA and mediates its connection to the other minor pilins subcomplex (PilVWXY1E)	
PD1615	*fimT2*	Prepilin type N-terminal cleavage/methylation domain		
PD1735	*fimT3*	Type IV fimbrial biogenesis protein		
**Pilus-associated adhesin**				
PD0023	*pilY1-1*	Type IV fimbrial biogenesis protein	TFP tip adhesin	3
PD1611	*pilY1-2*	Type IV fimbrial biogenesis protein		
PD0502	*pilY1-3*	Type IV fimbrial biogenesis protein		
**Secretin**				
PD1691	*pilQ*	Fimbrial assembly protein	Multimeric outer membrane secretin that forms gated pores from which TFP are extruded	1
**Pilotin**				
PD1623	*pilF*	Type IV fimbrial biogenesis protein/stability protein	Pilotin protein required for the outer membrane localization and assembly of the multimeric PilQ secretin	4
**Platform protein**				
PD1923	*pilC*	Fimbrial assembly protein	Platform protein that mediates TFP assembly through the polymerization and depolymerization ATPases	5

**Alignment protein**				
PD1695	*pilM*	Fimbrial assembly membrane protein	Alignment subcomplex that is involved in TFP assembly and stabilization by linking the outer membrane secretin pore subcomplex (PilF and PilQ) to the inner membrane motor subcomplex (PilB, PilC, PilD, PilT and PilU)	6
PD1694	*pilN*	Fimbrial assembly membrane protein		
PD1693	*pilO*	Fimbrial assembly membrane protein		
PD1692	*pilP*	Fimbrial assembly protein		
**Assembly ATPase**				
PD1927	*pilB*	Pilus biogenesis protein	TFP polymerization (assembly) ATPase; promotes TFP extension	1
**Retraction ATPase**				
PD1147	*pilT*	Type IV fimbrial biogenesis protein	TFP depolymerization (disassembly) ATPases; promote TFP retraction	1
PD1148	*pilU*	Type IV fimbrial biogenesis protein		
**Prepilin peptidase**				
PD1922	*pilD*	Type IV prepilin leader peptidase	Prepilin peptidase that cleaves the leader peptide of all pilins (major and minor) at the cytoplasmic milieu of the inner membrane and methylates the mature pilin	7,8
**Two-component regulatory system**				
PD1928	*pilR*	Two-component system, regulatory protein	Cytoplasmic response regulator that binds to the promoter of *pilA* (together with σ^{54}) to activate transcription of the major pilin	9,10
PD1929	*pilS*	Two-component system, sensor protein	Sensor kinase that interacts directly with PilA for pilin transcription autoregulation	11
**Chemotaxis protein**				
PD0845	*pilG*	Pilus protein	Chemosensory system composed by a transmembrane chemoreceptor (PilJ), a histidine kinase (PilL), a methylesterase (ChpB), response regulators (PilG and PilH) and coupling proteins (PilI and ChpC), which has been shown to affect twitching motility	12,13
PD1632	*pilH*	Regulatory protein		
PD0846	*pilI*	Type IV fimbrial biogenesis protein		
PD0847	*pilJ*	Type IV fimbrial biogenesis protein		
PD0848	*pilL*	Chemotaxis-related protein kinase		
PD0849	*chpB*	Chemotaxis response regulator protein		
PD0850	*chpC*	Chemotaxis protein		

**Signal transduction**				
PD1497	*pilZ*	Type IV fimbriae assembly protein	Regulatory protein associated with TFP extension in a possible c-di-GMP (3′,5′-cyclic-di-guanylate) -dependent manner	14,15

[a]Locus IDs and genome annotations are from the genome of *X. fastidiosa* subsp. *fastidiosa* reference strain Temecula1, GenBank Accession number AE009442.1. Genome annotation was visualized using Geneious (Biomatters Ltd.).
[b]Genes are organized by functional categories in the table.

References

1 Mattick, J. S. Type IV pili and twitching motility. *Annu Rev Microbiol* **56**, 289-314, doi:10.1146/annurev.micro.56.012302.160938 (2002).

2 Nguyen, Y. *et al. Pseudomonas aeruginosa* minor pilins prime type IVa pilus assembly and promote surface display of the PilY1 adhesin. *J Biol Chem* **290**, 601-611, doi:10.1074/jbc.M114.616904 (2015).

3 Alm, R. A., Hallinan, J. P., Watson, A. A. & Mattick, J. S. Fimbrial biogenesis genes of *Pseudomonas aeruginosa*: *pilW* and *pilX* increase the similarity of type 4 fimbriae to the GSP protein-secretion systems and *pilY1* encodes a gonococcal PilC homologuE. *Mol Microbiol* **22**, 161-173 (1996).

4 Koo, J. *et al.* PilF is an outer membrane lipoprotein required for multimerization and localization of the *Pseudomonas aeruginosa* Type IV pilus secretin. *J Bacteriol* **190**, 6961-6969, doi:10.1128/JB.00996-08 (2008).

5 Takhar, H. K., Kemp, K., Kim, M., Howell, P. L. & Burrows, L. L. The platform protein is essential for type IV pilus biogenesis. *J Biol Chem* **288**, 9721-9728, doi:10.1074/jbc.M113.453506 (2013).

6 Tammam, S. *et al.* PilMNOPQ from the *Pseudomonas aeruginosa* type IV pilus system form a transenvelope protein interaction network that interacts with PilA. *J Bacteriol* **195**, 2126-2135, doi:10.1128/JB.00032-13 (2013).

7 Strom, M. S., Nunn, D. N. & Lory, S. A single bifunctional enzyme, PilD, catalyzes cleavage and N-methylation of proteins belonging to the type IV pilin family. *Proc Natl Acad Sci U S A* **90**, 2404-2408 (1993).

8 Giltner, C. L., Habash, M. & Burrows, L. L. *Pseudomonas aeruginosa* minor pilins are incorporated into type IV pili. *J Mol Biol* **398**, 444-461, doi:10.1016/j.jmb.2010.03.028 (2010).

9 Ishimoto, K. Y. & Lory, S. Identification of pilR, which encodes a transcriptional activator of the *Pseudomonas aeruginosa* pilin gene. *J Bacteriol* **174**, 3514-3521 (1992).

10 Jin, S., Ishimoto, K. Y. & Lory, S. PilR, a transcriptional regulator of piliation in *Pseudomonas aeruginosa*, binds to a *cis*-acting sequence upstream of the pilin gene promoter. *Mol Microbiol* **14**, 1049-1057 (1994).

11 Kilmury, S. L. & Burrows, L. L. Type IV pilins regulate their own expression via direct intramembrane interactions with the sensor kinase PilS. *Proc Natl Acad Sci U S A* **113**, 6017-6022, doi:10.1073/pnas.1512947113 (2016).

12 Cursino, L. *et al.* Identification of an operon, Pil-Chp, that controls twitching motility and virulence in *Xylella fastidiosa*. *Mol Plant Microbe Interact* **24**, 1198-1206 (2011).

13 Darzins, A. Characterization of a *Pseudomonas aeruginosa* gene cluster involved in pilus biosynthesis and twitching motility: sequence similarity to the chemotaxis proteins of enterics and the gliding bacterium *Myxococcus xanthus*. *Mol Microbiol* **11**, 137-153 (1994).

14 Alm, R. A., Bodero, A. J., Free, P. D. & Mattick, J. S. Identification of a novel gene, *pilZ*, essential for type 4 fimbrial biogenesis in *Pseudomonas aeruginosa*. *J Bacteriol* **178**, 46-53 (1996).

15 Guzzo, C. R., Salinas, R. K., Andrade, M. O. & Farah, C. S. PILZ protein structure and interactions with PILB and the FIMX EAL domain: implications for control of type IV pilus biogenesis. *J Mol Biol* **393**, 848-866, doi:10.1016/j.jmb.2009.07.065 (2009).
