# Supplementary material for: Complete functional analysis of type IV pilus components of a reemergent plant pathogen reveals neofunctionalization of paralog genes
Source: PLoS Pathog. 2023 Feb 13;19(2):e1011154. doi: 10.1371/journal.ppat.1011154 (PMC9956873; doi:10.1371/journal.ppat.1011154)
Supplement: S2 Table — (PDF) [file ppat.1011154.s003.pdf]

Table S2. Genomic organization of *pil* and associated genes in *X. fastidiosa* TemeculaL.

Gene(s)[a]	Operon[b]	DNA strand
fimT1 (PD0019), *pilV1* (PD0020), *pilW1* (PD0021), *pilX1* (PD0022), *pilY1-1* (PD0023), *pilE1* (PD0024)	PD0019→PD0020→PD0021→PD0022→PD0023→PD0024	Leading
pilY1-3 (PD0502)	Not in operon	Leading
pilG (PD0845), *pilI* (PD0846), *pilJ* (PD0847), *cheA/pilL* (PD0848), *cheB/chpB* (PD0849), *cheW/chpC* (PD0850)	PD0845→PD0846→PD0847→PD0848→PD0849→PD0850	Leading
pilA3 PD1077	Not in operon	Lagging
pilT (PD1147)	Not in operon	Leading
pilU (PD1148)	Not in operon	Leading
pilZ (PD1497), **PD1498**, **PD1499**	PD1499→PD1498→PD1497	Lagging
pilE2 (PD1610), *pilY1-2* (PD1611)	PD1611→PD1610	Lagging
pilX2 (PD1612), *pilW2* (PD1613), *pilV2* (PD1614), *fimT2* (PD1615)	PD1615→PD1614→PD1613→PD1612	Lagging
pilF PD1623, **PD1622**	PD1623→PD1622	Lagging
pilH (PD1632), **PD1631**	PD1631→PD1632	Leading
pilQ (PD1691), *pilP* (PD1692), *pilO* (PD1693), *pilN* (PD1694), *pilM* (PD1695)	PD1695→PD1694→PD1693→PD1692→ PD1691	Lagging
fimT3 (PD1735)	Not in operon	Leading
**PD1921**, *pilD* (PD1922), *pilC* (PD1923)	PD1923→PD1922→PD1921	Lagging
pilA1 (PD1924)	Not in operon	Leading
pilA2 (PD1926)	Not in operon	Leading
pilB (PD1927)	Not in operon	Leading
pilR (PD1928)	Not in operon	Lagging
pilS (PD1929)	Not in operon	Lagging

[a]Genes in bold have no apparent effect on type IV pili and/or natural transformation. They are included in the list because they are located within the same operon of *pil* genes.
[b]Operon prediction was retrieved from elsewhere [1].

References

1 Parker, J. K., Chen, H., McCarty, S. E., Liu, L. Y. & De La Fuente, L. Calcium transcriptionally regulates the biofilm machinery of *Xylella fastidiosa* to promote continued biofilm development in batch cultures. *Environ Microbiol* **18**, 1620-1634, doi:10.1111/1462-2920.13242 (2016).
