# Supplementary material for: Complete functional analysis of type IV pilus components of a reemergent plant pathogen reveals neofunctionalization of paralog genes
Source: PLoS Pathog. 2023 Feb 13;19(2):e1011154. doi: 10.1371/journal.ppat.1011154 (PMC9956873; doi:10.1371/journal.ppat.1011154)
Supplement: S3 Table — (PDF) [file ppat.1011154.s004.pdf]

Table S3. Pearson's correlation results for the analyzed phenotypic traits of *X. fastidiosa*.

Phenotypic traits[a]	Recombination frequency	Twitching motility	Biofilm formation	Planktonic growth	Growth rate	Settling rate	Total viable CFU/ml[b]
**Recombination frequency**	-	**R^2 = 0.56** **P<0.001**	R^2 = -0.23 P = 0.15	R^2 = -0.14 P = 0.38	R^2 = 0.02 P = 0.89	R^2 = -0.11 P = 0.47	R^2 = -0.27 P = 0.09
**Twitching motility**	**R^2 = 0.56** **P<0.001**	-	**R^2 = -0.54** **P<0.001**	R^2 = -0.14 P = 0.38	R^2 = 0.09 P = 0.58	R^2 = -0.14 P = 0.38	R^2 = -0.10 P = 0.51
**Biofilm formation**	R^2 = -0.23 P = 0.15	**R^2 = -0.54** **P<0.001**	-	R^2 = -0.16 P = 0.30	**R^2 = -0.34** **P<0.05**	**R^2 = 0.59** **P<0.001**	R^2 = 0.01 P = 0.94
**Planktonic growth**	R^2 = -0.14 P = 0.38	R^2 = -0.14 P = 0.38	R^2 = -0.16 P = 0.30	-	**R^2 = 0.34** **P<0.05**	**R^2 = -0.55** **P<0.001**	R^2 = 0 P = 1.0
**Growth rate**	R^2 = 0.02 P = 0.89	R^2 = 0.09 P = 0.58	**R^2 = -0.34** **P<0.05**	**R^2 = 0.34** **P<0.05**	-	**R^2 = -0.51** **P<0.001**	**R^2 = -0.49** **P<0.005**
**Settling rate**	R^2 = -0.11 P = 0.47	R^2 = -0.14 P = 0.38	**R^2 = 0.59** **P<0.001**	**R^2 = -0.55** **P<0.001**	**R^2 = -0.51** **P<0.001**	-	R^2 = 0.24 P = 0.12
**Total viable CFU/ml[a]**	R^2 = -0.27 P = 0.09	R^2 = -0.10 P = 0.51	R^2 = 0.01 P = 0.94	R^2 = 0 P = 1.0	**R^2 = -0.49** **P<0.005**	R^2 = 0.24 P = 0.12	-

[a]Results are shown as correlation coefficients (R^2) and *p*-values (*P*). Results in bold show significant correlations (either positive or negative) among phenotypes.
[b]Total viable CFU/ml obtained in natural competence assays.
