# Supplementary material for: Complete functional analysis of type IV pilus components of a reemergent plant pathogen reveals neofunctionalization of paralog genes
Source: PLoS Pathog. 2023 Feb 13;19(2):e1011154. doi: 10.1371/journal.ppat.1011154 (PMC9956873; doi:10.1371/journal.ppat.1011154)
Supplement: S4 Table — (PDF) [file ppat.1011154.s005.pdf]

Table S4. DNA-binding probability of amino acid residues from FimT3.

Amino acid	Position	DNA-binding probability[a]
R (arginine)	160	0.8941
R (arginine)	162	0.8199
W (tryptophan)	81	0.8161
K (lysine)	177	0.784
N (asparagine)	180	0.7829
R (arginine)	93	0.7772
G (glycine)	159	0.7689
K (lysine)	94	0.7577
K (lysine)	179	0.7566
G (glycine)	84	0.7465
R (arginine)	123	0.7433
R (arginine)	54	0.7417
S (serine)	188	0.7412

[a]The DNA-binding probability of each amino acid residue from FimT3 of *X. fastidiosa* strain TemeculaL was calculated using the DRNApred webserver [1] and ranges from 0 to 1.0. Only amino acid residues with a DNA-binding probability above 0.74 are shown in the table.

References

1 Yan, J. & Kurgan, L. DRNApred, fast sequence-based method that accurately predicts and discriminates DNA- and RNA-binding residues. *Nucleic Acids Res* **45**, e84, doi:10.1093/nar/gkx059 (2017).
