# Supplementary material for: Complete functional analysis of type IV pilus components of a reemergent plant pathogen reveals neofunctionalization of paralog genes
Source: PLoS Pathog. 2023 Feb 13;19(2):e1011154. doi: 10.1371/journal.ppat.1011154 (PMC9956873; doi:10.1371/journal.ppat.1011154)
Supplement: S5 Table — (PDF) [file ppat.1011154.s006.pdf]

Table S5. Homologs of FimT3 are found in members of the Xanthomonadaceae family.

Bacterial species	Number of strains encoding FimT3[a]	Identical amino acids (%)[b]
Xylella		
X. fastidiosa	121	95.60 – 100
X. taiwanensis	9	80
Coralloluteibacterium		
C. stylophorae	1	37.13
Luteimonas		
L. aestuarii	1	36.05
L. arsenica	1	34.5
L. deserti	1	38.82
L. fraxinea	2	39.18
L. mephitis	1	38.01
L. padinae	1	35.5
L. sp.	9	33.73 – 39.18
L. terrae	1	36.47
L. terricola	1	36.75
L. yindakuii	1	36.31
Lysobacter		
L. sp.	1	28.92
Pseudoxanthomonas		
P. beigongshangi	1	41.38
P. composti	1	37.39
P. daejeonensis	2	40.48
P. gei	1	44.31
P. helianthi	1	27.88
P. japonensis	2	36.05 – 36.63
P. kaohsiungensis	1	40.83
P. koreensis	1	46.01
P. mexicana	7 (1 with no GRxR motif)	23.15 – 43.75
P. sacheonensis	1	40.23
P. sangjuensis	1	29.81
P. sp.	19	35.84 – 42.61
P. spadix	3	38.6 – 42.44
P. suwonensis	2	41.32
P. taiwanensis	1	41.92
P. winnipegensis	11	35.84 – 37.21
P. wuyuanensis	2	42.94
P. yeongjuensis	1	41.28
Rehaibacterium		
R. terrae	1	27.71
Stenotrophomonas		
S. acidaminiphila	7	34.52 – 35.71
S. humi	1	34.68
S. maltophilia	50	30.36 – 39.16
S. nitritireducens	1	34.73
S. pictorum	2	36.53
S. sp.	7	32.56 – 48.84
Vulcaniibacterium		
V. gelatinicum	2 (1 with no GRxR motif)	15.5 – 28.9
V. thermophilum	2	33.33
Xanthomonas		
X. albilineans	17	44.32

X. alfalfae	1	44.25
X. arboricola	96 (1 with no GRxR motif)	27.65 – 46.63
X. axonopodis	63 (26 with no GRxR motif)	27.54 – 44.83
X. bromi	2	45.4
X. campestris	128 (2 with no GRxR motif)	28.4 – 49.43
X. cannabis	3	43.1 – 44.77
X. cassavae	2	45.71
X. citri	31 (6 with no GRxR motif)	27.81 – 48.31
X. codiaei	1	43.02
X. dyei	3	29.34 – 42.44
X. euroxanthea	2	41.67 – 41.95
X. euvesicatoria	11 (1 with no GRxR motif)	28.4 – 45.8
X. floridensis	1	43.93
X. fragariae	56	34.91 – 35.5
X. hortorum	73	32.54 – 43.68
X. hyacinthi	1	37.95
X. maliensis	1	42.69
X. melonis	3	45.93
X. nasturtii	19	26.04 – 43.1
X. oryzae	404	42.53 – 43.79
X. perforans	19 (3 with no GRxR motif)	28.57 – 45.86
X. populi	1	41.95
X. prunicola	3	43.68
X. sacchari	5	44.32 – 44.51
X. sontii	5	46.02 – 46.82
X. sp.	34	27.81 – 48.86
X. theicola	2	46.82
X. translucens	69	51.7 – 52.27
X. vasicola	67	29.34 – 44.83
X. vesicatoria	11	28.24 – 44.19

[a]A total of 3,0001 genomes of bacterial species belonging to the Xanthomonadaceae family were downloaded from NCBI on May 25th, 2022. In the table only the number of genomes from bacterial species that encode homologs of FimT3 are shown.

[b]Sequence alignments to FimT3 of *X. fastidiosa* strain TemeculaL were produced using Clustal Omega (Clustal 12.1).
