# Supplementary material for: Complete functional analysis of type IV pilus components of a reemergent plant pathogen reveals neofunctionalization of paralog genes
Source: PLoS Pathog. 2023 Feb 13;19(2):e1011154. doi: 10.1371/journal.ppat.1011154 (PMC9956873; doi:10.1371/journal.ppat.1011154)
Supplement: S6 Table — (PDF) [file ppat.1011154.s007.pdf]

Table S6. Bacterial strains and plasmids used in this study.

Strain name in manuscript or plasmid	Genotype or description	Source
Xylella fastidiosa		
WT	Wild-type *X. fastidiosa* subsp. *fastidiosa* strain TemeculaL	1
Δ*pilA1*	*X. fastidiosa* strain TemeculaL with chromosomal *pilA1* (PD1924) deletion, Km^R	2
Δ*pilA2*	*X. fastidiosa* strain TemeculaL with chromosomal *pilA2* (PD1926) deletion, Km^R	This study
Δ*pilA3*	*X. fastidiosa* strain TemeculaL with chromosomal *pilA3* (PD1077) deletion, Km^R	This study
Δ*pilA1pilA2*	*X. fastidiosa* strain TemeculaL with chromosomal *pilA1* and *pilA2* (PD1924 and PD1926, respectively) deletion, Km^R, Cm^R	2
Δpi*lB*	*X. fastidiosa* strain TemeculaL with chromosomal *pilB* (PD1927) deletion, Km^R	This study
Δ*pilC*	*X. fastidiosa* strain TemeculaL with chromosomal *pilC* (PD1923) deletion, Km^R	This study
Δ*pilD*	*X. fastidiosa* strain TemeculaL with chromosomal *pilD* (PD1922) deletion, Km^R	This study
Δ*pilE1*	*X. fastidiosa* strain TemeculaL with chromosomal *pilE1* (PD0024) deletion, Km^R	This study
Δ*pilE2*	*X. fastidiosa* strain TemeculaL with chromosomal *pilE2* (PD1610) deletion, Km^R	This study
Δ*pilF*	*X. fastidiosa* strain TemeculaL with chromosomal *pilF* (PD1623) deletion, Km^R	This study
Δ*pilG*	*X. fastidiosa* strain TemeculaL with chromosomal *pilG* (PD0845) deletion, Km^R	This study
Δ*pilH*	*X. fastidiosa* strain TemeculaL with chromosomal *pilH* (PD1632) deletion, Km^R	This study
Δ*pilI*	*X. fastidiosa* strain TemeculaL with chromosomal *pilI* (PD0846) deletion, Km^R	This study
Δ*pilJ*	*X. fastidiosa* strain TemeculaL with chromosomal *pilJ* (PD0847) deletion, Km^R	This study
Δ*pilL*	*X. fastidiosa* strain TemeculaL with chromosomal *pilL* (PD0848) deletion, Km^R	This study
Δ*pilM*	*X. fastidiosa* strain TemeculaL with chromosomal *pilM* (PD1695) deletion, Km^R	This study
Δ*pilN*	*X. fastidiosa* strain TemeculaL with chromosomal *pilN* (PD1694) deletion, Km^R	This study
Δ*pilO*	*X. fastidiosa* strain TemeculaL with chromosomal *pilO* (PD1693) deletion, Km^R	This study
Δ*pilP*	*X. fastidiosa* strain TemeculaL with chromosomal *pilP* (PD1694) deletion, Km^R	This study
Δ*pilQ*	*X. fastidiosa* strain TemeculaL with chromosomal *pilQ* (PD1691) deletion, Km^R	This study
Δ*pilR*	*X. fastidiosa* strain TemeculaL with chromosomal *pilR* (PD1928) deletion, Km^R	This study
Δ*pilS*	*X. fastidiosa* strain TemeculaL with chromosomal *pilS* (PD1929) deletion, Km^R	This study
Δ*pilT*	*X. fastidiosa* strain TemeculaL with chromosomal *pilT* (PD1147) deletion, Km^R	This study
Δ*pilU*	*X. fastidiosa* strain TemeculaL with chromosomal *pilU* (PD1148) deletion, Km^R	This study
Δ*pilV1*	*X. fastidiosa* strain TemeculaL with chromosomal *pilV1* (PD0020) deletion, Km^R	This study

Δ*pilV2*	*X. fastidiosa* strain TemeculaL with chromosomal *pilV2* (PD1614) deletion, Km^{R}	This study
Δ*pilW1*	*X. fastidiosa* strain TemeculaL with chromosomal *pilW1* (PD0021) deletion, Km^{R}	This study
Δ*pilW2*	*X. fastidiosa* strain TemeculaL with chromosomal *pilW2* (PD1613) deletion, Km^{R}	This study
Δ*pilX1*	*X. fastidiosa* strain TemeculaL with chromosomal *pilX1* (PD0022) deletion, Km^{R}	This study
Δ*pilX2*	*X. fastidiosa* strain TemeculaL with chromosomal *pilX2* (PD1612) deletion, Km^{R}	This study
Δ*pilY1-1*	*X. fastidiosa* strain TemeculaL with chromosomal *pilY1-1* (PD0023) deletion, Km^{R}	This study
Δ*pilY1-2*	*X. fastidiosa* strain TemeculaL with chromosomal *pilY1-2* (PD1611) deletion, Km^{R}	This study
Δ*pilY1-3*	*X. fastidiosa* strain TemeculaL with chromosomal *pilY1-3* (PD0502) deletion, Km^{R}	This study
Δ*pilZ*	*X. fastidiosa* strain TemeculaL with chromosomal *pilZ* (PD1497) deletion, Km^{R}	This study
Δ*fimT1*	*X. fastidiosa* strain TemeculaL with chromosomal *fimT1* (PD0019) deletion, Km^{R}	This study
Δ*fimT2*	*X. fastidiosa* strain TemeculaL with chromosomal *fimT2* (PD1615) deletion, Km^{R}	This study
Δ*fimT3*	*X. fastidiosa* strain TemeculaL with chromosomal *fimT3* (PD1735) deletion, Km^{R}	This study
Δ*chpB*	*X. fastidiosa* strain TemeculaL with chromosomal *chpB* (PD0849) deletion, Km^{R}	This study
Δ*chpC*	*X. fastidiosa* strain TemeculaL with chromosomal *chpC* (PD0850) deletion, Km^{R}	This study
Escherichia coli		
Dh5α	*fhuA2 Δ(argF-lacZ)U169 phoA glnV44 Φ80 Δ(lacZ)M15 gyrA96 recA1 relA1 endA1 thi-1 hsdR17*	New England Biolabs
EAM1	DH5α derivative; Sp^{r} St^{r}*attP*$_{HK022}$::(P$_{LlacO-1}$-PD1607) Expresses the *X. fastidiosa* subsp. *fastidiosa* strain Temecula1 DNA methylase	[3]
BL21(DE3)	*fhuA2 [lon] ompT gal (λ DE3) [dcm] ΔhsdS λ DE3 = λ sBamHIo ΔEcoRI-B int::(lacI::PlacUV5::T7 gene1) i21 Δnin5*	New England Biolabs
Dh5α-pUC4K	*E. coli* Dh5α bearing the pUC4K plasmid	[2]
EAM1-pAX1-Cm	*E. coli* EAM1 bearing the pAX1-Cm plasmid	[4]
Dh5α-pHIS-Parallel1-*fimT1s*	*E. coli* Dh5α bearing the pHIS-Parallel1-*fimT1s* plasmid	This study
Dh5α-pHIS-Parallel1-*fimT2s*	*E. coli* Dh5α bearing the pHIS-Parallel1-*fimT2s* plasmid	This study
Dh5α-pHIS-Parallel1-*fimT3s*	*E. coli* Dh5α bearing the pHIS-Parallel1-*fimT3s* plasmid	This study
Dh5α-pHIS-Parallel1-*fimT3s*-R160AR162A	*E. coli* Dh5α bearing the pHIS-Parallel1-*fimT3s*-R160AR162A plasmid	This study
BL21(DE3)-pHIS-Parallel1-*fimT1s*	*E. coli* BL21(DE3) bearing the pHIS-Parallel1-*fimT1s* plasmid	This study
BL21(DE3)-pHIS-Parallel1-*fimT2s*	*E. coli* BL21(DE3) bearing the pHIS-Parallel1-*fimT2s* plasmid	This study

BL21(DE3)-pHIS-Parallel1-*fimT3s*	*E. coli* BL21(DE3) bearing the pHIS-Parallel1-*fimT3s* plasmid	This study
BL21(DE3)-pHIS-Parallel1-*fimT3s*-R160AR162A	*E. coli* BL21(DE3) bearing the pHIS-Parallel1-*fimT3s*-R160AR162A plasmid	This study
Dh5α-pLas16S	*E. coli* Dh5α bearing the pLas16S plasmid	[5]
**Plasmids**		
pUC4K	Donor of kanamycin resistance cassette, Km^R Amp^R	[6]
pAX1-Cm	pGEM-T derivative; contains multiple cloning site Plasmid that recombines into the neutral site 1 (NS1) of *X. fastidiosa* and inserts a chloramphenicol resistance cassette	[7]
pHIS-Parallel1	Cloning vector, f1 ori *lacI* T7 promoter (P_{T7}) Amp^R	[8]
pHIS-Parallel1-*fimT1s*	Amp^R, P_{T7}-*fimT1s*	This study
pHIS-Parallel1-*fimT2s*	Amp^R, P_{T7}-*fimT2s*	This study
pHIS-Parallel1-*fimT3s*	Amp^R, P_{T7}-*fimT3s*	This study
pHIS-Parallel1-*fimT3s*-R160AR162A	Amp^R, P_{T7}-*fimT3s*-R160AR162A	This study
pLas16S[a]	Plasmid standard to quantify the population of '*Candidatus* Liberibacter asiaticus' as genome equivalents through qPCR	[5]
pGEM-T	Cloning vector, f1 ori *lacZ* Amp^R	Promega

Km^R – kanamycin-resistant; Cm^R – chloramphenicol-resistant; Amp^R – ampicillin-resistant.
[a]In this study, pLas16S was used for EMSA in agarose gel with FimT3s.

References

1 Potnis, N. *et al.* Patterns of inter- and intrasubspecific homologous recombination inform eco-evolutionary dynamics of *Xylella fastidiosa*. *ISME J* **13**, 2319-2333, doi:10.1038/s41396-019-0423-y (2019).

2 Kandel, P. P., Chen, H. & De La Fuente, L. A short protocol for gene knockout and complementation in *Xylella fastidiosa* shows that one of the type IV pilin paralogs (PD1926) is needed for twitching while another (PD1924) affects pilus number and location. *Appl Environ Microbiol* **84**, e01167-01118 (2018).

3 Matsumoto, A. & Igo, M. M. Species-specific type II restriction-modification system of *Xylella fastidiosa* Temecula1. *Appl Environ Microbiol* **76**, 4092-4095, doi:10.1128/AEM.03034-09 (2010).

4 Kandel, P. P., Almeida, R. P. P., Cobine, P. A. & De La Fuente, L. Natural competence rates are variable among *Xylella fastidiosa* strains and homologous recombination occurs in vitro between subspecies *fastidiosa* and *multiplex*. *Mol Plant Microbe Interact* **30**, 589-600, doi:10.1094/MPMI-02-17-0053-R (2017).

5 Parker, J. K. *et al.* Viability of '*Candidatus* Liberibacter asiaticus' prolonged by addition of citrus juice to culture medium. *Phytopathology* **104**, 15-26 (2014).

6 Vieira, J. & Messing, J. The pUC plasmids, an M13mp7-derived system for insertion mutagenesis and sequencing with synthetic universal primers. *Gene* **19**, 259-268 (1982).

7 Matsumoto, A., Young, G. M. & Igo, M. M. Chromosome-based genetic complementation system for *Xylella fastidiosa*. *Appl Environ Microbiol* **75**, 1679-1687, doi:10.1128/AEM.00024-09 (2009).

8 Sheffield, P., Garrard, S. & Derewenda, Z. Overcoming expression and purification problems of RhoGDI using a family of "Parallel" expression vectors. *Protein Expr. Purif.* **15**, 34-39 (1999).
