# Supplementary material for: Complete functional analysis of type IV pilus components of a reemergent plant pathogen reveals neofunctionalization of paralog genes
Source: PLoS Pathog. 2023 Feb 13;19(2):e1011154. doi: 10.1371/journal.ppat.1011154 (PMC9956873; doi:10.1371/journal.ppat.1011154)
Supplement: S7 Table — (PDF) [file ppat.1011154.s008.pdf]

**Table S7.** List of PCR primers and qPCR primers and probe used in this study.

| Name | Sequence (5'–3') | Purpose or description | Amplicon size (bp) | Source |
|---|---|---|---|---|
| **Site-directed mutagenesis of genes of interest[a]** | | | | |
| pilA2-Up F | CCTCAGGAATCATCCGTAACC | Amplify the upstream region of *pilA2* (PD1926) | 839 | This study |
| pilA2-Up R | **GTCAGCAACACCTTCTTCACGAGGCAGAC**GATGAATCCTTAAATAGCGTTGGTAAG | | | |
| pilA2-Down F | **CATCAGAGATTTTGAGACACAACGTGGC**TTAACACCAGCAACAACACGATTC | Amplify the downstream region of *pilA2* (PD1926) | 909 | This study |
| pilA2-Down R | GGTGATGCCGACAAGATTGGCTG | | | |
| pilA3-Up F | CAGTAGCCCTATCCGTGAATGTGTC | Amplify the upstream region of *pilA3* (PD1077) | 823 | This study |
| pilA3-Up R | **GTCAGCAACACCTTCTTCACGAGGCAGAC**AAAAATTCCCCTAATCTTTGAAAGTG | | | |
| pilA3-Down F | **CATCAGAGATTTTGAGACACAACGTGGC**CCGAACGAGTAATGAGCGCCGATG | Amplify the downstream region of *pilA3* (PD1077) | 859 | This study |
| pilA3-Down R | CCATCACAGACCTATGCGATACTG | | | |
| pilB-Up F | GATAACAACGCAGGCCAAGGTG | Amplify the upstream region of *pilB* (PD1927) | 973 | This study |
| pilB-Up R | **GTCAGCAACACCTTCTTCACGAGGCAGAC**GAAAAGTTCTCTGGTTACTCTGC | | | |
| pilB-Down F | **CATCAGAGATTTTGAGACACAACGTGGC**GCATCAACAACACCTGTTAATGAC | Amplify the downstream region of *pilB* (PD1927) | 1,034 | This study |
| pilB-Down R | CCTTGGTGAATCAGGAGTTGG | | | |
| pilC-Up F | CAGTGCAGCCGGAAGGTCTCAG | Amplify the upstream region of *pilC* (PD1923) | 845 | This study |
| pilC-Up R | **GTCAGCAACACCTTCTTCACGAGGCAGAC**TGCTGTTCTCCCATCCACCGTC | | | |
| pilC-Down F | **CATCAGAGATTTTGAGACACAACGTGGC**AACGTTATGGCATTTCTTGATC | Amplify the downstream region of *pilC* (PD1923) | 835 | This study |
| pilC-Down R | CCATCCACCAACTGCCAGATAAG | | | |
| pilD-Up F | GTATGCGTACGATGGTCAATC | Amplify the upstream region of *pilD* (PD1922) | 958 | This study |
| pilD-Up R | **GTCAGCAACACCTTCTTCACGAGGCAGAC**AACGTTTTATCCAACGACAGAAG | | | |
| pilD-Down F | **CATCAGAGATTTTGAGACACAACGTGGC**GTGAGGTTGGAGTTGATGAGTGTC | Amplify the downstream region of *pilD* (PD1922) | 851 | This study |
| pilD-Down R | GCAACTCATACATCCATACAC | | | |
| pilE1-Up F | CTGTCTGGTGTATTTCCCTGGTC | Amplify the upstream region of *pilE1* (PD0024) | 874 | This study |
| pilE1-Up R | **GTCAGCAACACCTTCTTCACGAGGCAGAC**GGAAGTTACATCATTCACGAACATC | | | |
| pilE1-Down F | **CATCAGAGATTTTGAGACACAACGTGGC**GGTGATCTGATGTTTGGAGTGCTTG | Amplify the downstream region of *pilE1* (PD0024) | 952 | This study |
| pilE1-Down R | CAGGAGTCATCCGTCGTCTTTCG | | | |

| | | | | |
|---|---|---|---|---|
| pilE2-Up F | GCAATCTCTGGAAGTTCAACCTG | Amplify the upstream region of *pilE2* (PD1610) | 912 | This study |
| pilE2-Up R | **GTCAGCAACACCTTCTTCACGAGGCAGAC**ACGTTCCCACTTTAGGATCACCATC | | | |
| pilE2-Down F | **CATCAGAGATTTTGAGACACAACGTGGC**CGTGTTCCCGCATTGCTTTTAGTTG | Amplify the downstream region of *pilE2* (PD1610) | 893 | This study |
| pilE2-Down R | CTCATAGACGAACACGAGTAGG | | | |
| pilF-Up F | CAAGGTTTTAACCGCAATCTGAC | Amplify the upstream region of *pilF* (PD1623) | 855 | This study |
| pilF-Up R | **GTCAGCAACACCTTCTTCACGAGGCAGAC**GACTAAGCAGCCAGATAAAATC | | | |
| pilF-Down F | **CATCAGAGATTTTGAGACACAACGTGGC**TGTATTGTGATCAGTGATTTCG | Amplify the downstream region of *pilF* (PD1623) | 814 | This study |
| pilF-Down R | GTTCCCAGATTCTTGCACTTCCACC | | | |
| pilG-Up F | CAGATAGCGTTGCGCTATTGC | Amplify the upstream region of *pilG* (PD0845) | 869 | This study |
| pilG-Up R | **GTCAGCAACACCTTCTTCACGAGGCAGAC**AGCGCTCTGAATCTAAATACTGTG | | | |
| pilG-Down F | **CATCAGAGATTTTGAGACACAACGTGGC**CCTGACTGTTCATCTGATGCGTTTCC | Amplify the downstream region of *pilG* (PD0845) | 803 | This study |
| pilG-Down R | CAACTGCTGCGACAACACCTG | | | |
| pilH-Up F | GAAGTGATAGTTCGCGCGTTATG | Amplify the upstream region of *pilH* (PD1632) | 1,003 | This study |
| pilH-Up R | **GTCAGCAACACCTTCTTCACGAGGCAGAC**GTCTGCTTCAGCAGTTTAGTGTG | | | |
| pilH-Down F | **CATCAGAGATTTTGAGACACAACGTGGC**GGCACACCATAACGAGAAACCGGAC | Amplify the downstream region of *pilH* (PD1632) | 977 | This study |
| pilH-Down R | GTTATGTTGACTCCCTTCTCTG | | | |
| pilI-Up F | CTACCAGGGTGCACATAATGAAG | Amplify the upstream region of *pilI* (PD0846) | 845 | This study |
| pilI-Up R | **GTCAGCAACACCTTCTTCACGAGGCAGAC**CAGATGAACAGTCAGGTTAAACAG | | | |
| pilI-Down F | **CATCAGAGATTTTGAGACACAACGTGGC**TTGGCGGTCCCGTCTTGCATATTTAG | Amplify the downstream region of *pilI* (PD0846) | 977 | This study |
| pilI-Down R | GTTTATCACGTACCGAGCCAACC | | | |
| pilJ-Up F | GGTGTGAGGTGGTTACCGCTATTG | Amplify the upstream region of *pilJ* (PD0847) | 884 | This study |
| pilJ-Up R | **GTCAGCAACACCTTCTTCACGAGGCAGAC**TCAGGCAGCAGCCTGTCTAAATTCAG | | | |
| pilJ-Down F | **CATCAGAGATTTTGAGACACAACGTGGC**TTGAATGCTTCTCGGCTTGGAAAGG | Amplify the downstream region of *pilJ* (PD0847) | 928 | This study |
| pilJ-Down R | GGAAGCATCGACATGGAGCAATG | | | |

| | | | | |
|---|---|---|---|---|
| pilL-Up F | CGATAGCCGACGCGATTAACTGTG | Amplify the upstream region of *pilL* (PD0848) | 922 | This study |
| pilL-Up R | **GTCAGCAACACCTTCTTCACGAGGCAGAC**TCAAGCTGGCAATTTGAAGTTGGCTG | | | |
| pilL-Down F | **CATCAGAGATTTTGAGACACAACGTGGC**TAGCGTCCAAGGTAAGTTGGTTGC | Amplify the downstream region of *pilL* (PD0848) | 920 | This study |
| pilL-Down R | GATGCCCAGATTATCCCGAAGCAC | | | |
| pilM-Up F | CGTGCATCGGTATTGCTTTTGC | Amplify the upstream region of *pilM* (PD1695) | 1,016 | This study |
| pilM-Up R | **GTCAGCAACACCTTCTTCACGAGGCAGAC**GGGCACTTTTAAGACAGGAACATATC | | | |
| pilM-Down F | **CATCAGAGATTTTGAGACACAACGTGGC**ATGGCCAGAATTAACTTATTGCCCTG | Amplify the downstream region of *pilM* (PD1695) | 987 | This study |
| pilM-Down R | CTTACTAGGCAACTGCCGTAACATC | | | |
| pilN-Up F | GCTTCAAGGTGGAACACTATGCTG | Amplify the upstream region of *pilN* (PD1694) | 994 | This study |
| pilN-Up R | **GTCAGCAACACCTTCTTCACGAGGCAGAC**TCAGTCAAAACTCCTCAAAGCCAGAC | | | |
| pilN-Down F | **CATCAGAGATTTTGAGACACAACGTGGC**ATGAGTAAGAATTCGTTTAAATTGAG | Amplify the downstream region of *pilN* (PD1694) | 1,153 | This study |
| pilN-Down R | GTTCGACGCGATCTTCATTCACC | | | |
| pilO-Up F | CTATGCTGGCAGTGAGTACAAC | Amplify the upstream region of *pilO* (PD1693) | 920 | This study |
| pilO-Up R | **GTCAGCAACACCTTCTTCACGAGGCAGAC**TCAATTGTCCTCTGACAGCG | | | |
| pilO-Down F | **CATCAGAGATTTTGAGACACAACGTGGC**ATGAGTACAAAAACCCTCAAAAAAATAG | Amplify the downstream region of *pilO* (PD1693) | 912 | This study |
| pilO-Down R | GATCTGGCGTTGCAAGACAGTC | | | |
| pilP-Up F | GATAGCAGGGCTCTGCCGTATG | Amplify the upstream region of *pilP* (PD1692) | 853 | This study |
| pilP-Up R | **GTCAGCAACACCTTCTTCACGAGGCAGAC**TCATGGTCCATCCTTCTTGC | | | |
| pilP-Down F | **CATCAGAGATTTTGAGACACAACGTGGC**ATGATTGATTTAAGGGATAG | Amplify the downstream region of *pilP* (PD1692) | 822 | This study |
| pilP-Down R | CAAATTACTTTCTCATCACGGC | | | |
| pilQ-Up F | GAGGTGCTGAAGCAGATGTTAC | Amplify the upstream region of *pilQ* (PD1691) | 967 | This study |
| pilQ-Up R | **GTCAGCAACACCTTCTTCACGAGGCAGAC**TTAATCGTCGAACGAAAGCGC | | | |
| pilQ-Down F | **CATCAGAGATTTTGAGACACAACGTGGC**ATTTCGTCAATGCACTGCTTTATATCTC | Amplify the downstream region of *pilQ* (PD1691) | 897 | This study |
| pilQ-Down R | CAACTCGAAACGCTTGGCACAAC | | | |

| | | | | |
|---|---|---|---|---|
| pilR-Up F | CGTTGACTTTGTTGGCGCTTGG | Amplify the upstream region of *pilR* (PD1928) | 900 | This study |
| pilR-Up R | **GTCAGCAACACCTTCTTCACGAGGCAGAC**GAATGCCAAGATAACGCAGCAGAC | | | |
| pilR-Down F | **CATCAGAGATTTTGAGACACAACGTGGC**TGAGAGGAAGCAGGCAACATAGC | Amplify the downstream region of *pilR* (PD1928) | 937 | This study |
| pilR-Down R | GGTATCGACAAACTCGGTTACG | | | |
| pilS-Up F | CCATCCAGTGCGTACAAATCC | Amplify the upstream region of *pilS* (PD1929) | 924 | This study |
| pilS-Up R | **GTCAGCAACACCTTCTTCACGAGGCAGAC**GTCACGGTGTCCTAAGGCAGAAGAG | | | |
| pilS-Down F | **CATCAGAGATTTTGAGACACAACGTGGC**GTGCCCCGGTATTTCATTCACATTC | Amplify the downstream region of *pilS* (PD1929) | 881 | This study |
| pilS-Down R | GTATGCGCACCGGTGAAACTC | | | |
| pilT-Up F | ACATCATCTGGCACATCAG | Amplify the upstream region of *pilT* (PD1147) | 925 | This study |
| pilT-Up R | **GTCAGCAACACCTTCTTCACGAGGCAGAC**TTGCATAGACGACGAATGG | | | |
| pilT-Down F | **TCAGAGATTTTGAGACACAACGTGGC**TTACCGCACTATCACACATAAG | Amplify the downstream region of *pilT* (PD1147) | 903 | This study |
| pilT-Down R | GCTTGATTGGCGTTGTTG | | | |
| pilU-Up F | GATTCTGGTGACTGGGCCGACTG | Amplify the upstream region of *pilU* (PD1148) | 855 | This study |
| pilU-Up R | **GTCAGCAACACCTTCTTCACGAGGCAGAC**CGGGTGTTGCTCCTTTGCTGCTATG | | | |
| pilU-Down F | **CATCAGAGATTTTGAGACACAACGTGGC**ATCTATTATGAGGTTATATAATC | Amplify the downstream region of *pilU* (PD1148) | 879 | This study |
| pilU-Down R | GTCGATGGTCGCTATGAATTGC | | | |
| pilV1-Up F | GGAACGAAGGAGGTCTCAAAGG | Amplify the upstream region of *pilV1* (PD0020) | 858 | This study |
| pilV1-Up R | **GTCAGCAACACCTTCTTCACGAGGCAGAC**AACAGTGTTTTATTCGTCGTTAGG | | | |
| pilV1-Down F | **CATCAGAGATTTTGAGACACAACGTGGC**GTGAACCGTCGGTTTGCCCTGCAAC | Amplify the downstream region of *pilV1* (PD0020) | 885 | This study |
| pilV1-Down R | CAAGGCAACTCTAACCGCAATGAC | | | |
| pilV2-Up F | GCTGGCAACGTCAAGGAAGAAG | Amplify the upstream region of *pilV2* (PD1614) | 861 | This study |
| pilV2-Up R | **GTCAGCAACACCTTCTTCACGAGGCAGAC**TCATTGACACGCGCCCTTCTCCACC | | | |
| pilV2-Down F | **CATCAGAGATTTTGAGACACAACGTGGC**CGATGTATTCCTCACGCTTAGCGCGTC | Amplify the downstream region of *pilV2* (PD1614) | 911 | This study |
| pilV2-Down R | CAACGGGCACGATACAGTGATG | | | |
| pilW1-Up F | CAGTGCCAACGAGTTAGTTGCTTC | Amplify the upstream region of *pilW1* (PD0021) | 936 | This study |
| pilW1-Up R | **GTCAGCAACACCTTCTTCACGAGGCAGAC**TCACAGTTTACTCCTCACTTCAAAAATTTG | | | |

| | | | | |
|---|---|---|---|---|
| pilW1-Down F | **CATCAGAGATTTTGAGACACAACGTGGC**ATGAGGATGACTCACCTCGGGCCGTACAG | Amplify the downstream region of *pilW1* (PD0021) | 973 | This study |
| pilW1-Down R | CGTAATTGGCTTGAGGCCTTGGAG | | | |
| pilW2-Up F | GCTTATGGTCGTTGTTGCTGTCG | Amplify the upstream region of *pilW2* (PD1613) | 988 | This study |
| pilW2-Up R | **GTCAGCAACACCTTCTTCACGAGGCAGAC**CGTCATAACACACTAGTCAGATTG | | | |
| pilW2-Down F | **CATCAGAGATTTTGAGACACAACGTGGC**TGATGAAGTTACGGGCATTTGTCG | Amplify the downstream region of *pilW2* (PD1613) | 1,069 | This study |
| pilW2-Down R | CATACCCGTCATGCGACACACTAGC | | | |
| pilX1-Up F | GAAGTGAGGAGTAAACTGTGAACC | Amplify the upstream region of *pilX1* (PD0022) | 997 | This study |
| pilX1-Up R | **GTCAGCAACACCTTCTTCACGAGGCAGAC**TCATAAATTTCTGTTCCGTAAGCTG | | | |
| pilX1-Down F | **CATCAGAGATTTTGAGACACAACGTGGC**TTGGATTTCTTCAAGATATCGAAATG | Amplify the downstream region of *pilX1* (PD0022) | 931 | This study |
| pilX1-Down R | CACCATCAATCAAGTAGCTGTG | | | |
| pilX2-Up F | GTAGTGCGTCTGATTGGAATCC | Amplify the upstream region of *pilX2* (PD1612) | 843 | This study |
| pilX2-Up R | **GTCAGCAACACCTTCTTCACGAGGCAGAC**CATCAGTTCCCGTATAAGCGATTG | | | |
| pilX2-Down F | **CATCAGAGATTTTGAGACACAACGTGGC**GCAATGAAAAAAAACGTTCTGAAGTG | Amplify the downstream region of *pilX2* (PD1612) | 849 | This study |
| pilX2-Down R | CGTAACTCATCGGCAACAGAAC | | | |
| pilY1-1-Up F | CGATCATCACCGAATTGCGTGCAG | Amplify the upstream region of *pilY1-1* (PD0023) | 959 | This study |
| pilY1-1-Up R | **GTCAGCAACACCTTCTTCACGAGGCAGAC**TTCGATATCTTGAAGAAATCC | | | |
| pilY1-1-Down F | **CATCAGAGATTTTGAGACACAACGTGGC**CGATGTTCGTGAATGATGTAAC | Amplify the downstream region of *pilY1-1* (PD0023) | 864 | This study |
| pilY1-1-Down R | CTACGCAAACGTGTTGCCATACAG | | | |
| pilY1-2-Up F | GGAGTGGAGTCCTTGCAATTTC | Amplify the upstream region of *pilY1-2* (PD1611) | 988 | This study |
| pilY1-2-Up R | **GTCAGCAACACCTTCTTCACGAGGCAGAC**ATTGAGCGGCGGCACCACGTTGCTC | | | |
| pilY1-2-Down F | **CATCAGAGATTTTGAGACACAACGTGGC**TGGTGATCCTAAAGTGGGAACGTG | Amplify the downstream region of *pilY1-2* (PD1611) | 1,045 | This study |
| pilY1-2-Down R | CGATTCAATGTCCAGCTGCATCC | | | |
| pilY1-3-Up F | GGTGTATTAGGGATAGTGGAGTTC | Amplify the upstream region of *pilY1-3* (PD0502) | 918 | This study |
| pilY1-3-Up R | **GTCAGCAACACCTTCTTCACGAGGCAGAC**CAAATCTTGTTCCTTCATCCTTGCTC | | | |

| | | | | |
|---|---|---|---|---|
| pilY1-3-Down F | **CATCAGAGATTTTGAGACACAACGTGGC**TCCACAAAAAAAGAGATGGATAGAG | Amplify the downstream region of *pilY1-3* (PD0502) | 868 | This study |
| pilY1-3-Down R | GCTTCCACACCTCATTATTCTGTC | | | |
| pilZ-Up F | GAAGGATTAGGGCAACGTGCGGTTTC | Amplify the upstream region of *pilZ* (PD1497) | 883 | This study |
| pilZ-Up R | **GTCAGCAACACCTTCTTCACGAGGCAGACT**ACTCCTTACATTCTGCTCAGCATTC | | | |
| pilZ-Down F | **CATCAGAGATTTTGAGACACAACGTGGC**TTGCTGTTGTGCTTGATGTGTTGCTTG | Amplify the downstream region of *pilZ* (PD1497) | 1,028 | This study |
| pilZ-Down R | GAACGAAGATGCTCTAGCACTGC | | | |
| fimT1-Up F | GCTTGGTGCGTATTACTCAGGCCATC | Amplify the upstream region of *fimT1* (PD0019) | 909 | This study |
| fimT1-Up R | **GTCAGCAACACCTTCTTCACGAGGCAGAC**ATTTAGATACATATGCCACG | | | |
| fimT1-Down F | **CATCAGAGATTTTGAGACACAACGTGGC**AACACTGTTATGTCCATCGCAAGC | Amplify the downstream region of *fimT1* (PD0019) | 879 | This study |
| fimT1-Down R | CAGAATGCGGATTGCGTCTGTTCC | | | |
| fimT2-Up F | CATCGCTGCCGAATCGCCAAATC | Amplify the upstream region of *fimT2* (PD1615) | 880 | This study |
| fimT2-Up R | **GTCAGCAACACCTTCTTCACGAGGCAGAC**CTAAACGCTCCACAGCGTTAATGTC | | | |
| fimT2-Down F | **CATCAGAGATTTTGAGACACAACGTGGC**GTGTCAATGAAAAAGTGTTCCAATC | Amplify the downstream region of *fimT2* (PD1615) | 832 | This study |
| fimT2-Down R | CATCCGCATTGAGAAACAACGAACG | | | |
| fimT3-Up F | GTCTGACCGGTAAGCGGATCGAAC | Amplify the upstream region of *fimT3* (PD1735) | 884 | This study |
| fimT3-Up R | **GTCAGCAACACCTTCTTCACGAGGCAGAC**ACCGATAGTCTCGACAATGTTTAGG | | | |
| fimT3-Down F | **CATCAGAGATTTTGAGACACAACGTGGC**ACACCATCGTATTCACATGACATTTG | Amplify the downstream region of *fimT3* (PD1735) | 900 | This study |
| fimT3-Down R | GCATAAGGATTACGCGCTTTCACC | | | |
| chpB-Up F | GTCAGAGCACCTTTGCAGTTC | Amplify the upstream region of *chpB* (PD0849) | 845 | This study |
| chpB-Up R | **GTCAGCAACACCTTCTTCACGAGGCAGAC**CTTACCTTGGACGCTATCATTCTC | | | |
| chpB-Down F | **CATCAGAGATTTTGAGACACAACGTGGC**TATGTAAGGAAACTACCTTCAATG | Amplify the downstream region of *chpB* (PD0849) | 1,042 | This study |
| chpB-Down R | CAACTACGCTCCGATGAGGTAAAGC | | | |

| | | | | |
|---|---|---|---|---|
| chpC-Up F | CGAATTGATTGCTCGTCGTCAAGG | Amplify the upstream region of *chpC* (PD0850) | 984 | This study |
| chpC-Up R | **GTCAGCAACACCTTCTTCACGAGGCAGAC**TGAAGGTAGTTTCCTTACATATCAATG | | | |
| chpC-Down F | **CATCAGAGATTTTGAGACACAACGTGGC**CATGTGGTGGGATGAATGTGTTTTG | Amplify the downstream region of *chpC* (PD0850) | 857 | This study |
| chpC-Down R | CCAGATTGTCCAGATGGTAAGAG | | | |
| Km-F | GTCTGCCTCGTGAAG | Amplify the kanamycin resistance cassette | 1,202 | [1] |
| Km-R | AAGCCACGTTGTGT | | | |
| **Confirmation of site-directed mutagenesis of genes of interest** | | | | |
| pilA2-Fconf | CACTTTGATCGAGCTGATGATC | Amplify internal sequence of *pilA2* (PD1926) | 328 | This study |
| pilA2-Rconf | GTTGTTATCAGCGATGCGGGTCAAG | | | |
| pilA2Up-Fconf | CAGGTCACTCCATCTGCCTTACC | Amplify internal sequence of upstream region of *pilA2* (PD1926) and the kanamycin resistance cassette | 972 | This study |
| pilA3-Fconf | CAAGGCTTCACCCTGTTAGAGG | Amplify internal sequence of *pilA3* (PD1077) | 410 | This study |
| pilA3-Rconf | CTTGAGCCATGCAAACCTACATCG | | | |
| pilA3UP-Fconf | CGTCAATAACGCGAACAGCATC | Amplify internal sequence of upstream region of *pilA3* (PD1077) and the kanamycin resistance cassette | 1,119 | This study |
| pilB-Fconf | CTACTGGATGTGTCCGCATTCG | Amplify internal sequence of *pilB* (PD1927) | 493 | This study |
| pilB-Rconf | CAGCCTTCAGCAGACCATCAATCC | | | |
| pilBUp-Fconf | CCATCCATGCAGCGTTTCCATCTG | Amplify internal sequence of upstream region of *pilB* (PD1927) and the kanamycin resistance cassette | 1,130 | This study |
| pilC-Fconf | CATCACGCCTCTTGTGGTTAAG | Amplify internal sequence of *pilC* (PD1923) | 649 | This study |
| pilC-Rconf | CAACACCAACCTATCCAACAG | | | |
| pilCUp-Fconf | GTTGTACTCTTCCCATCAGCAATC | Amplify internal sequence of upstream region of *pilC* (PD1923) and the kanamycin resistance cassette | 1,096 | This study |
| pilD-Fconf | GGAAAACATTCCGGTGCTTAGC | Amplify internal sequence of *pilD* (PD1922) | 500 | This study |
| pilD-Rconf | CAAGCCAAACAGAGCCCAATACC | | | |
| pilDUp-Fconf | CCAACTTTATGCAGCGGTGGTG | Amplify internal sequence of upstream region of *pilD* (PD1922) and the kanamycin resistance cassette | 1,100 | This study |

| | | | | |
|---|---|---|---|---|
| pilE1-Fconf | GATGATTGTGGTGGTGATCGTG | Amplify internal sequence of *pilE1* (PD0024) | 305 | This study |
| pilE1-Rconf | GTGTGAGCCTGCCACAGTGATC | | | |
| pilE1Up-Fconf | CCAGGTACAGATTGTTGATGAG G | Amplify internal sequence of upstream region of *pilE1* (PD0024) and the kanamycin resistance cassette | 1,044 | This study |
| pilE2-Fconf | GTTGATGGTTGTGGTCGCTGTC | Amplify internal sequence of *pilE2* (PD1610) | 301 | This study |
| pilE2-Rconf | GCACTTGTCGTTTTGCTGAGTG C | | | |
| pilE2Up-Fconf | GGTATGTCGATTTGGTGGTACA G | Amplify internal sequence of upstream region of *pilE2* (PD1610) and the kanamycin resistance cassette | 1,024 | This study |
| pilF-Fconf | GTTTACAACGTGCGTGATGATG | Amplify internal sequence of *pilF* (PD1623) | 388 | This study |
| pilF-Rconf | CACAACCGCCGGAATTGGCTAA AG | | | |
| pilFUp-Fconf | CAAACTAATGCGGCAATTTGAC | Amplify internal sequence of upstream region of *pilF* (PD1623) and the kanamycin resistance cassette | 878 | This study |
| pilG-Fconf | GTCTTAGGGTGATGGTCATTG | Amplify internal sequence of *pilG* (PD0845) | 305 | This study |
| pilG-Rconf | CAGATATTGTTCGGAACCAACC AC | | | |
| pilGUp-Fconf | CAGCATTGACTGGAGGATCTTT AC | Amplify internal sequence of upstream region of *pilG* (PD0845) and the kanamycin resistance cassette | 1,042 | This study |
| pilH-Fconf | GCATTTTGATCGTCGAGGACTC | Amplify internal sequence of *pilH* (PD1632) | 313 | This study |
| pilH-Rconf | GAGAAAGGCTTGGTGATGTAGG | | | |
| pilHUp-Fconf | CTGGCTTTGGACATCTTCAGTG | Amplify internal sequence of upstream region of *pilH* (PD1632) and the kanamycin resistance cassette | 1,100 | This study |
| pilI-Fconf | GTGTTGGCTATCGTATTGGAAG | Amplify internal sequence of *pilI* (PD0846) | 252 | This study |
| pilI-Rconf | CGATAACCAGTGCTGCATTATC | | | |
| pilIUp-Fconf | GCGTCTGCTATTGCAGTACACA G | Amplify internal sequence of upstream region of *pilI* (PD0846) and the kanamycin resistance cassette | 1,021 | This study |
| pilJ-Fconf | GCTGATCGCTTCGTGAGTAATG TG | Amplify internal sequence of *pilJ* (PD0847) | 557 | This study |
| pilJ-Rconf | CCAACAATCGACACTACAGACA GC | | | |

| | | | | |
|---|---|---|---|---|
| pilJUp-Fconf | CCTGACTGTTCATCTGATGCGTTTC | Amplify internal sequence of upstream region of *pilJ* (PD0847) and the kanamycin resistance cassette | 1,072 | This study |
| pilL-Fconf | GCTTGATGAAGAGCAGGGTAATC | Amplify internal sequence of *pilL* (PD0848) | 464 | This study |
| pilL-Rconf | GGATTTCACGCAGTACACTGTCC | | | |
| pilLUp-Fconf | CGAGATTGCGGCTAGTATCGAAC | Amplify internal sequence of upstream region of *pilL* (PD0848) and the kanamycin resistance cassette | 1,236 | This study |
| pilM-Fconf | GCAGGATTTGGAAGCTCAGATAG | Amplify internal sequence of *pilM* (PD1695) | 408 | This study |
| pilM-Rconf | CCATAGCGATGCATTACTTCGTC | | | |
| pilMUp-Fconf | GTCAATGCCGCTGTGTTCGTAG | Amplify internal sequence of upstream region of *pilM* (PD1695) and the kanamycin resistance cassette | 1,049 | This study |
| pilN-Fconf | GGTCAGTGGTCAGAATGATCG | Amplify internal sequence of *pilN* (PD1694) | 439 | This study |
| pilN-Rconf | CTTATCTGGCGACTCTGGCTTGG | | | |
| pilNUp-Fconf | CAACAACACGGAGATGGTCCAAG | Amplify internal sequence of upstream region of *pilN* (PD1694) and the kanamycin resistance cassette | 1,188 | This study |
| pilO-Fconf | GTCATCATGGCTTGGTTCCTC | Amplify internal sequence of *pilO* (PD1693) | 377 | This study |
| pilO-Rconf | GGTAAAGAAGCCACACCACTGAC | | | |
| pilOUp-Fconf | CACATTGGTGGGTATCCTCGTTTC | Amplify internal sequence of upstream region of *pilO* (PD1693) and the kanamycin resistance cassette | 1,105 | This study |
| pilP-Fconf | GTCGGTTTGATTGTTCTCTTGG | Amplify internal sequence of *pilP* (PD1692) | 433 | This study |
| pilP-Rconf | GTTCGACGCGATCTTCATTCAC | | | |
| pilPUp-Fconf | GCAGCCATTAAAGCAGCAGTTG | Amplify internal sequence of upstream region of *pilP* (PD1692) and the kanamycin resistance cassette | 970 | This study |
| pilQ-Fconf | CGTATCGACGCTAAGCCTATGG | Amplify internal sequence of *pilQ* (PD1691) | 567 | This study |
| pilQ-Rconf | CATACCTTTGGCTTCTGTCAGTGC | | | |

| | | | | |
|---|---|---|---|---|
| pilQUp-Fconf | GAGAATCGTACTGCTGATGCTG | Amplify internal sequence of upstream region of *pilQ* (PD1691) and the kanamycin resistance cassette | 1,096 | This study |
| pilR-Fconf | CGTATCAGAACGGCAGCTAATCTG | Amplify internal sequence of *pilR* (PD1928) | 400 | This study |
| pilR-Rconf | CTTGACTACGCGCCACTTTAGC | | | |
| pilRUp-Fconf | CGATGAATACCGCCAGACATTG | Amplify internal sequence of upstream region of *pilR* (PD1928) and the kanamycin resistance cassette | 1,137 | This study |
| pilS-Fconf | CTTGTGGAATACATCTGGACTG | Amplify internal sequence of *pilS* (PD1929) | 642 | This study |
| pilS-Rconf | CCTTTGATCGCCTTTGTCAAG | | | |
| pilSUp-Fconf | CTGTTTCACTTCGTCGGTTGTC | Amplify internal sequence of upstream region of *pilS* (PD1929) and the kanamycin resistance cassette | 992 | This study |
| pilT-Fconf | GTGATGACATTGGACGAACTCG | Amplify internal sequence of *pilT* (PD1147) | 719 | This study |
| pilT-Rconf | CTTTATCCTTGGCGTATTCACG | | | |
| pilTUp-Fconf | CCTTCTTTACTATCTCCTGAGC | Amplify internal sequence of upstream region of *pilT* (PD1147) and the kanamycin resistance cassette | 951 | This study |
| pilU-Fconf | CTCAATGGCATCAATGATCAAC | Amplify internal sequence of *pilU* (PD1148) | 365 | This study |
| pilU-Rconf | GATTCAGCGACAGATCCATCAG | | | |
| pilUUp-Fconf | GATCATCTCGCAAATGTTGTTG | Amplify internal sequence of upstream region of *pilU* (PD1148) and the kanamycin resistance cassette | 940 | This study |
| pilV1-Fconf | CGTTAGTTTGATCGAAGTGCTG | Amplify internal sequence of *pilV1* (PD0020) | 323 | This study |
| pilV1-Rconf | CATCACAAGAGATCGAACCACAG | | | |
| pilV1Up-Fconf | GAACGAAGGAGGTCTCAAAGGAAAGC | Amplify internal sequence of upstream region of *pilV1* (PD0020) and the kanamycin resistance cassette | 1,353 | This study |
| pilV2-Fconf | GTGTTCCAATCATCGTTGCCATTG | Amplify internal sequence of *pilV2* (PD1614) | 381 | This study |
| pilV2-Rconf | CCATTCGTCGCAAGATTCACC | | | |
| pilV2Up-Fconf | CAGACAGTGTTTGCGCTGCACTG | Amplify internal sequence of upstream region of *pilV2* (PD1614) and the kanamycin resistance cassette | 1,111 | This study |

| | | | | |
|---|---|---|---|---|
| pilW1-Fconf | GAGATTCCGCCACAGAATGGTC | Amplify internal sequence of *pilW1* (PD0021) | 405 | This study |
| pilW1-Rconf | CATACCACTGCACTGCACGCAATTC | | | |
| pilW1Up-Fconf | CCAACACACGGATGGCTAACAAC | Amplify internal sequence of upstream region of *pilW1* (PD0021) and the kanamycin resistance cassette | 1,124 | This study |
| pilW2-Fconf | CGTCATGGACTGCTTACCGTCTTTC | Amplify internal sequence of *pilW2* (PD1613) | 408 | This study |
| pilW2-Rconf | GCAGTACCAGAACATCACTTCC | | | |
| pilW2Up-Fconf | CAGTTGTTCGTGTCAGTGAAGG | Amplify internal sequence of upstream region of *pilW2* (PD1613) and the kanamycin resistance cassette | 1,189 | This study |
| pilX1-Fconf | GCTAACCTCCGTGATCGCAGTTTG | Amplify internal sequence of *pilX1* (PD0022) | 409 | This study |
| pilX1-Rconf | CATTGCTAGACGGATCGCTGCTTCG | | | |
| pilX1Up-Fconf | CTCAAGGCGTCAGAGGAACAGAC | Amplify internal sequence of upstream region of *pilX1* (PD0022) and the kanamycin resistance cassette | 1,148 | This study |
| pilX2-Fconf | GTTGTGCTGGTGGTGTTGGTG | Amplify internal sequence of *pilX2* (PD1612) | 350 | This study |
| pilX2-Rconf | CTGAATACAGTGATACGGAAC | | | |
| pilX2Up-Fconf | GTTTGAACGAGCGCGGGATTCC | Amplify internal sequence of upstream region of *pilX2* (PD1612) and the kanamycin resistance cassette | 940 | This study |
| pilY1-1-Fconf | GCTAATCTGGCCGACACCTACAC | Amplify internal sequence of *pilY1-1* (PD0023) | 479 | This study |
| pilY1-1-Rconf | GAATCGTAAGGTTGGCCCTCTGTG | | | |
| pilY1-1Up-Fconf | CTGTTCTGCGCACGACGTTACTTC | Amplify internal sequence of upstream region of *pilY1-1* (PD0023) and the kanamycin resistance cassette | 1,042 | This study |
| pilY1-2-Fconf | CACATCGCGTATGGATGCTTTGC | Amplify internal sequence of *pilY1-2* (PD1611) | 541 | This study |
| pilY1-2-Rconf | CTGGTATGAGCCAGTCAACAGAC | | | |
| pilY1-2Up-Fconf | CAAGTTGCAACCATGCAGGAG | Amplify internal sequence of upstream region of *pilY1-2* (PD1611) and the kanamycin resistance cassette | 1,044 | This study |

| | | | | |
|---|---|---|---|---|
| pilY1-3-Fconf | CAGTGATGGATCGTGCTTATCC | Amplify internal sequence of *pilY1-3* (PD0502) | 552 | This study |
| pilY1-3-Rconf | GAGTAGTCGCCTTAGCATCCTCAG | | | |
| pilY1-3Up-Fconf | GCATCTCTGGATTGTCAGCATCG | Amplify internal sequence of upstream region of *pilY1-3* (PD0502) and the kanamycin resistance cassette | 1,187 | This study |
| pilZ-Fconf | CAGGGTATTCTGTCGCTCACC | Amplify internal sequence of *pilZ* (PD1497) | 326 | This study |
| pilZ-Rconf | GTGTGCGTTGGCTTATCTGAG | | | |
| pilZUp-Fconf | GCATTGCTGAAGACGCTTGAG | Amplify internal sequence of upstream region of *pilZ* (PD1497) and the kanamycin resistance cassette | 1,088 | This study |
| fimT1-Fconf | CAATTCTTGCGGGCCTTGCTTATC | Amplify internal sequence of *fimT1* (PD0019) | 396 | This study |
| fimT1-Rconf | GGTTGTTAGCCATCCGTGTGTTGG | | | |
| fimT1Up-Fconf | CAAAGGTGTTGATGACCCGATG | Amplify internal sequence of upstream region of *fimT1* (PD0019) and the kanamycin resistance cassette | 1,088 | This study |
| fimT2-Fconf | GCTTATGGTCGTTGTTGCTGTCG | Amplify internal sequence of *fimT2* (PD1615) | 367 | This study |
| fimT2-Rconf | GTTCGATGCACGCCGTAAGCCATGC | | | |
| fimT2Up-Fconf | GTGAAGAACGCAAGGATGCTTATTAC | Amplify internal sequence of upstream region of *fimT2* (PD1615) and the kanamycin resistance cassette | 1,014 | This study |
| fimT3-Fconf | CACGATGGCGCTAATCGCATTATTG | Amplify internal sequence of *fimT3* (PD1735) | 398 | This study |
| fimT3-Rconf | CATTGTTGCAGAAGCGGATGCTG | | | |
| fimT3Up-Fconf | CAACACATCAGAACGTGACAGC | Amplify internal sequence of upstream region of *fimT3* (PD1735) and the kanamycin resistance cassette | 1,090 | This study |
| chpB-Fconf | GATTGCTCGTCGTCAAGGTTG | Amplify internal sequence of *chpB* (PD0849) | 569 | This study |
| chpB-Rconf | GATCAACTGGCAAAGTGGAGACTC | | | |
| chpBUp-Fconf | CACTGGTGATGGTTGTGTGATTG | Amplify internal sequence of upstream region of *chpB* (PD0849) and the kanamycin resistance cassette | 1,034 | This study |

| | | | | |
|---|---|---|---|---|
| chpC-Fconf | CGAACGAGTGCTATTACCAAATGC | Amplify internal sequence of *chpC* (PD0850) | 357 | This study |
| chpC-Rconf | GCTTGAGTTTCACTAAGAAGTACAC | | | |
| chpCUp-Fconf | GCATTACCCGCTGGTTTCAAACATC | Amplify internal sequence of upstream region of *chpC* (PD0850) and the kanamycin resistance cassette | 1,020 | This study |
| Km-Rconf | GATTCAGTCGTCACTCATGGTG | Pairs with "Up-Fconf" primers to amplify internal sequence of upstream region of the gene of interest and the kanamycin resistance cassette | - | [2] |
| **Confirmation of pAX1-Cm recombination into the NS1 region of *X. fastidiosa* TemeculaL** | | | | |
| Cm-F | AATCAGCGACACTGAATACGG | Amplify the chloramphenicol resistance cassette | 1,119 | [1] |
| Cm-R | TCACTTATTCAGGCGTAGCAC | | | |
| **qPCR primers and probe used to quantify the *X. fastidiosa* population** | | | | |
| HL5 Forward | AAGGCAATAAACGCGCACTA | Quantify the *X. fastidiosa* population as genome equivalents | 221 | [3] |
| HL6 Reverse | GGTTTTGCTGACTGGCAACA | | | |
| HLp (probe) | 6FAM-TGGCAGGCAGCAACGATACGGCT-QSY | | | |
| **Cloning into pHIS-Parallel1[b]** | | | | |
| fimT1s-pHIS-F | CATG**CCATGG**ATAATGGTGAGCGCTTG (NcoI) | Amplify *fimT1s* to clone into pHIS-Parallel1 | 459 | This study |
| fimT1s-pHIS-R | CCG**CTCGAG**TTATTCGTCGTTAGG (XhoI) | | | |
| fimT2s-pHIS-F | CATG**CCATGG**ATCGCTCGAATCGAGTG (NcoI) | Amplify *fimT2s* to clone into pHIS-Parallel1 | 429 | This study |
| fimT2s-pHIS-R | CCG**CTCGAG**TCATTGACACGC (XhoI) | | | |
| fimT3s-pHIS-F | CATG**CCATGG**ATAAACAAACTCTGGAAC (NcoI) | Amplify *fimT3s* to clone into pHIS-Parallel1 | 519 | This study |
| fimT3s-pHIS-R | CCG**CTCGAG**TTATTGGAATGGTGTTTC (XhoI) | | | |
| **Amino acid exchanges in FimT3s (cloned into pHIS-Parallel1)** | | | | |
| FimT3_R160A R162A-F | GAGTATTAAGTATCCGTTGCGAGGCAATTGCGCCTAAATTGTTGATCACGATGA | Exchange the arginine amino acid residues at positions 160 and 162 of FimT3s cloned into pHIS-Parallel1 by alanine residues | 5,859 | This study |
| FimT3_R160A R162A-R | TCATCGTGATCAACAATTTAGGCGCAATTGCCTCGCAACGGATACTTAATACTC | | | |
| **Sanger sequencing** | | | | |
| T7 promoter - forward | TAATACGACTCACTATAGGG | Amplify the gene cloned downstream of the T7 promoter in pHIS-Parallel1 for Sanger sequencing | variable | Eurofins Genomics LLC |

[a]Nucleotides in bold indicate the 5' extended region of each primer that is homologous to the kanamycin resistance cassette sequence to allow fusion of chromosomal sequences to the marker by overlap-extension PCR.
[b]Nucleotides in bold indicate the restriction sites of the endonucleases (specified within parenthesis) used for cloning into pHIS-Parallel1.

References

1 Kandel, P. P., Chen, H. & De La Fuente, L. A short protocol for gene knockout and complementation in *Xylella fastidiosa* shows that one of the type IV pilin paralogs (PD1926) is needed for twitching while another (PD1924) affects pilus number and location. *Appl Environ Microbiol* **84**, e01167-01118 (2018).

2 Gluck-Thaler, E. *et al.* Repeated gain and loss of a single gene modulates the evolution of vascular plant pathogen lifestyles. *Sci Adv* **6**, eabc4516 (2020).

3 Francis, M., Lin, H., Rosa, J. C.-L., Doddapaneni, H. & Civerolo, E. L. Genome-based PCR primers for specific and sensitive detection and quantification of *Xylella fastidiosa*. *European Journal of Plant Pathology* **115**, 203-213, doi:10.1007/s10658-006-9009-4 (2006).
