# Supplementary figures and images for: Complete functional analysis of type IV pilus components of a reemergent plant pathogen reveals neofunctionalization of paralog genes
Source: PLoS Pathog. 2023 Feb 13;19(2):e1011154. doi: 10.1371/journal.ppat.1011154 (PMC9956873; doi:10.1371/journal.ppat.1011154)

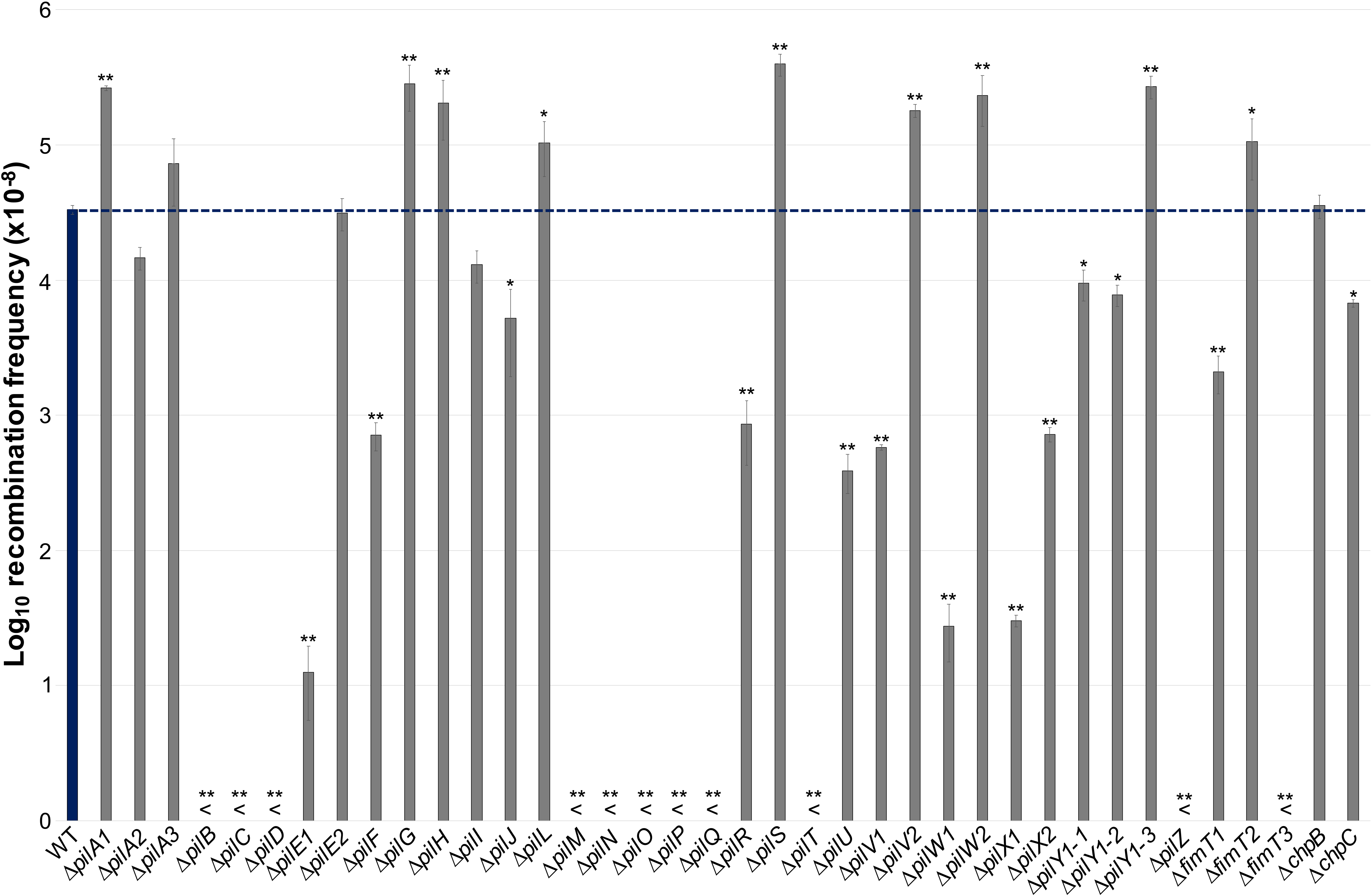

Supplement: S1 Fig — Quantification of natural transformation was performed in PD3 plates by applying 1 μg of the pAX1-Cm plasmid to equivalent numbers of recipient cells of each X. fastidiosa strain to generate chloramphenicol-resistant (CmR) mutations. Total viable cells and transformants were counted and results are expressed as the ratio of recipient cells transformed. WT is highlighted in blue, and the dashed blue line indicates the mean value of recombination frequency for the WT. Data represent means and standard errors. * and ** indicate significant difference (P<0.05 and P<0.005, respectively) of recombination frequency through natural competence in comparison to the WT as determined using Student’s t-test (n = three to 21 independent replicates with two internal replicates each). “<” indicates below detection limit, which was 10−7. Mutant strains below the detection limit were considered non-recombinant. (TIF) [file ppat.1011154.s009.tif]

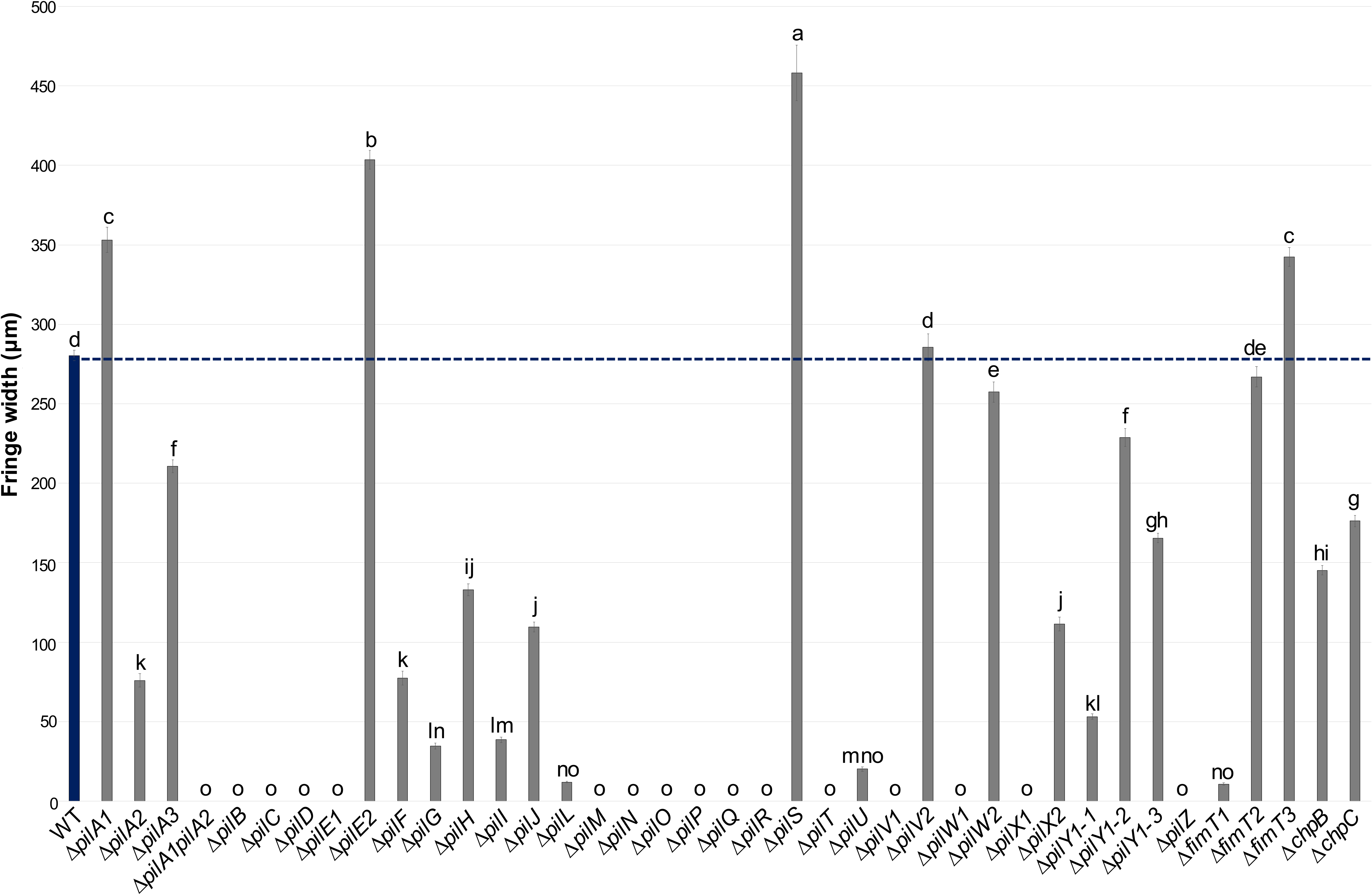

Supplement: S2 Fig — Twitching motility was determined by spotting cells of each strain in PW without BSA plates and measuring the movement fringe width after 4 days of growth at 28°C. WT is highlighted in blue, and the dashed blue line indicates the mean value of fringe width for the WT. Data represent means and standard errors. Different letters on top of bars indicate significant difference as analyzed by ANOVA followed by Tukey’s HSD multiple comparisons of means (P<0.05; n = three to 14 independent replicates with eight to 48 internal replicates each). The detection limit was 10 μm. Mutant strains below the detection limit were considered non-motile. (TIF) [file ppat.1011154.s010.tif]

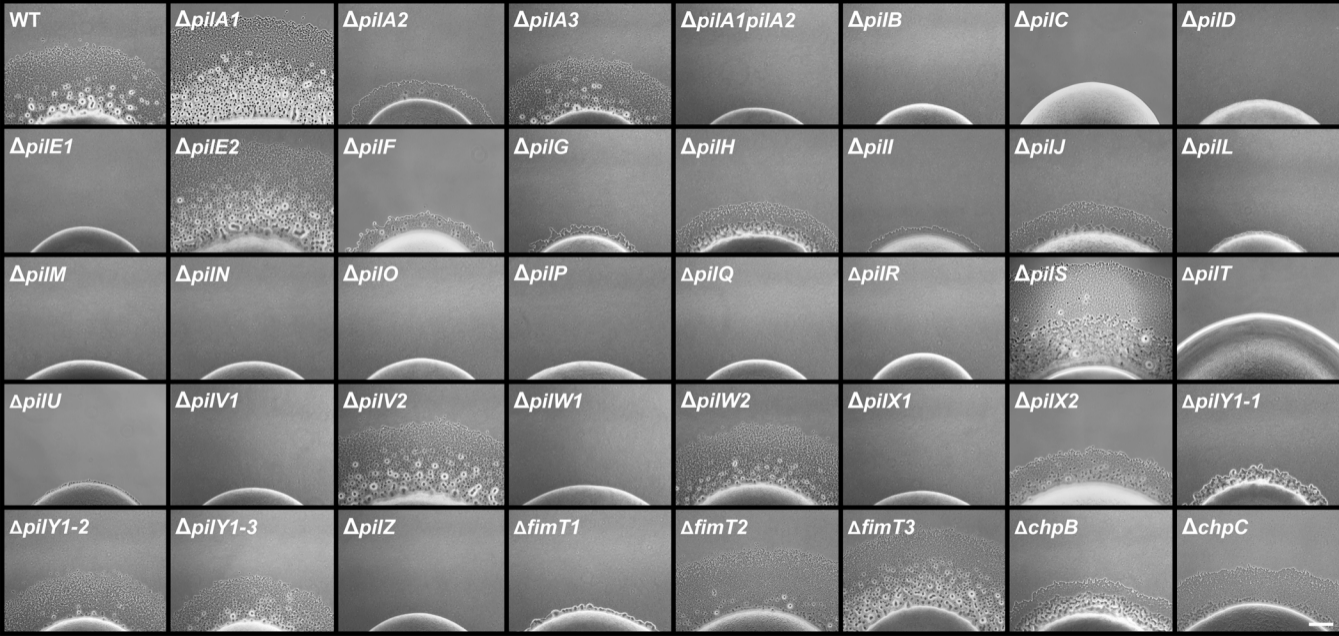

Supplement: S3 Fig — The assay was performed as described in S2 Fig. Similar events were captured in three to 14 independent experiments. Images were captured at 10× magnification. Scale bar (right lower panel), 100 μm. (PDF) [file ppat.1011154.s011.pdf]

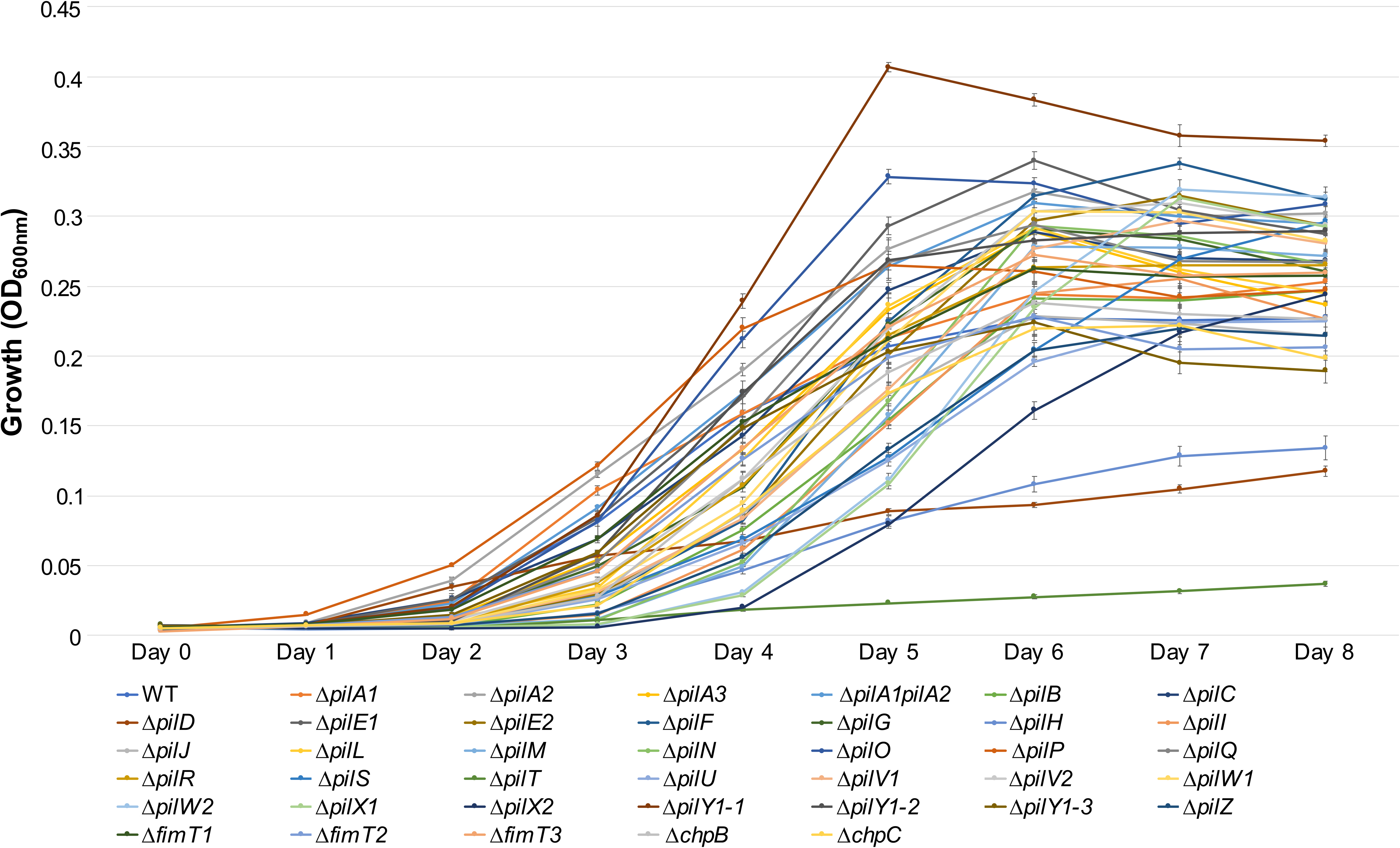

Supplement: S4 Fig — Growth curves were generated by culturing bacteria in PD3 broth within 96-well plates and measuring the optical density at 600 nm (OD600nm) values each day for 8 days. Data represent means and standard errors (n = three to 15 independent replicates, with eight internal replicates each). (TIF) [file ppat.1011154.s012.tif]

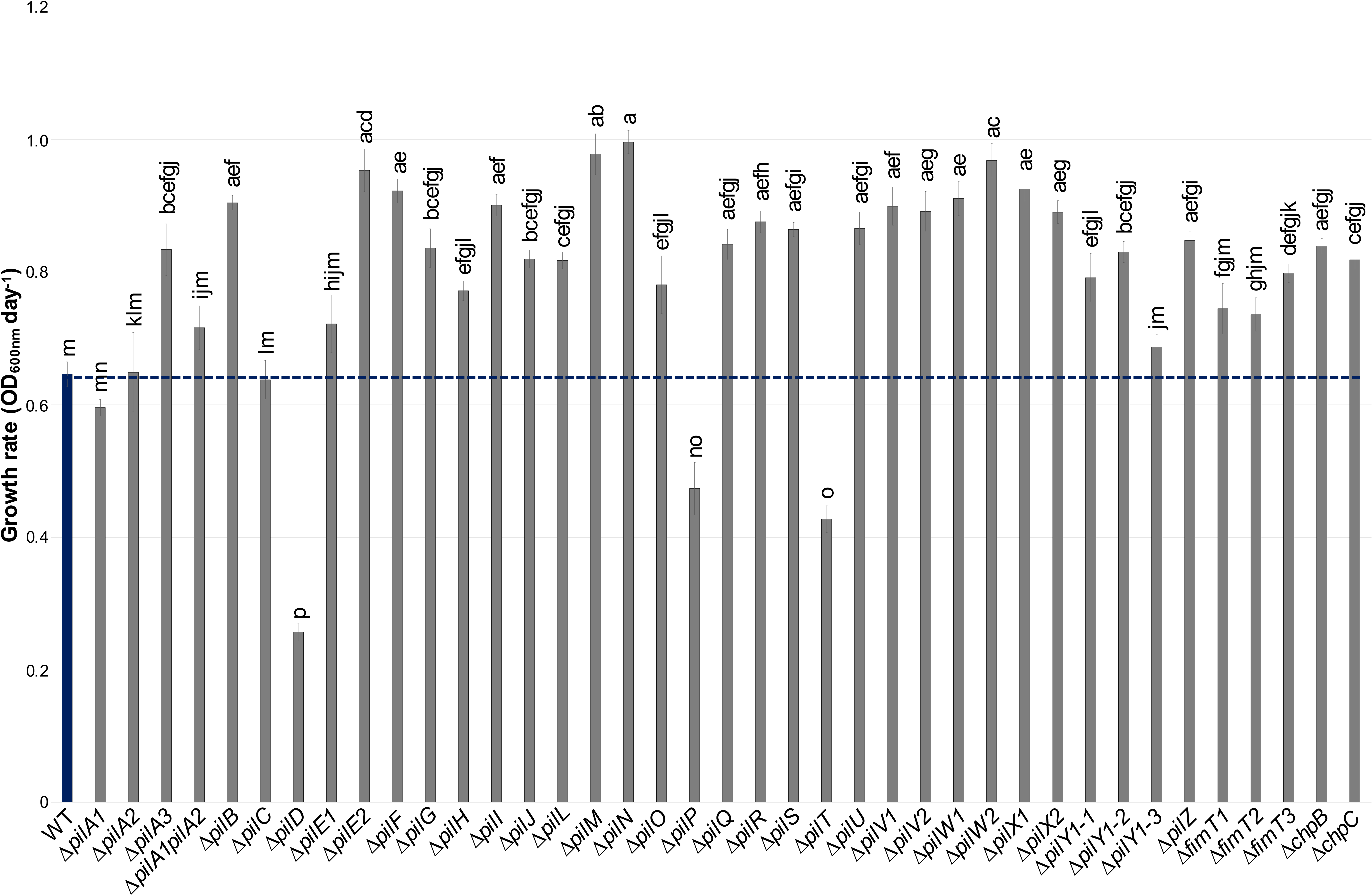

Supplement: S5 Fig — Growth rate was calculated from the growth curve at the exponential growth phase (2 to 6 days post inoculation). WT is highlighted in blue, and the dashed blue line indicates the mean value of growth rate for the WT. Data represent means and standard errors. Different letters on top of bars indicate significant difference as analyzed by ANOVA followed by Tukey’s HSD multiple comparisons of means (P<0.05; n = three to 15 independent replicates with eight internal replicates each). (TIF) [file ppat.1011154.s013.tif]

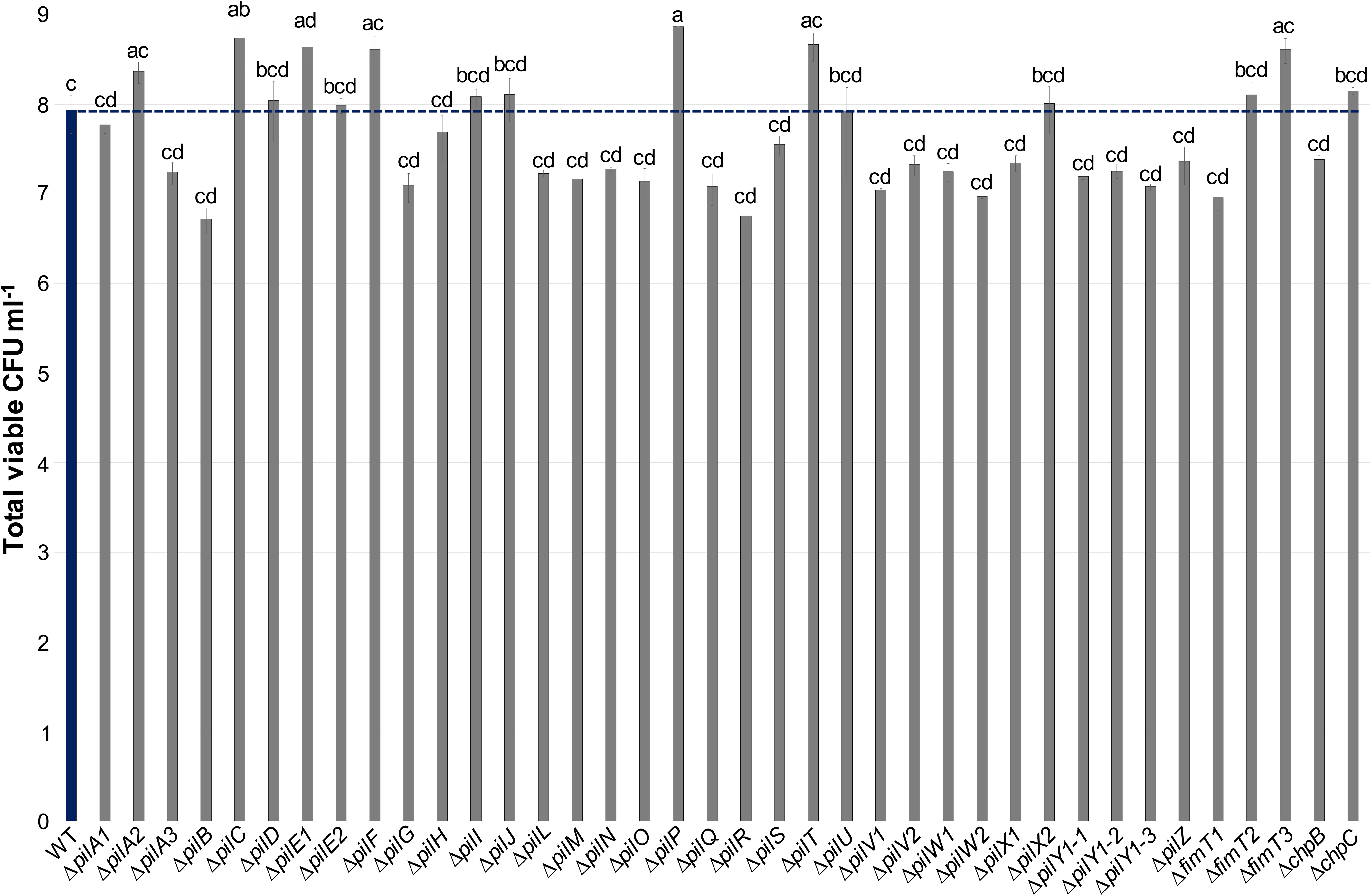

Supplement: S6 Fig — Total viable CFUs of each X. fastidiosa strain obtained during natural competence assays described in S1 Fig are shown here. WT is highlighted in blue, and the dashed blue line indicates the mean value of the total number of viable CFU/ml obtained during natural competence assays for the WT. Data represent means and standard errors. Different letters on top of bars indicate significant difference as analyzed by ANOVA followed by Tukey’s HSD multiple comparisons of means (P<0.05; n = three to 21 independent replicates with two internal replicates each). (TIF) [file ppat.1011154.s014.tif]

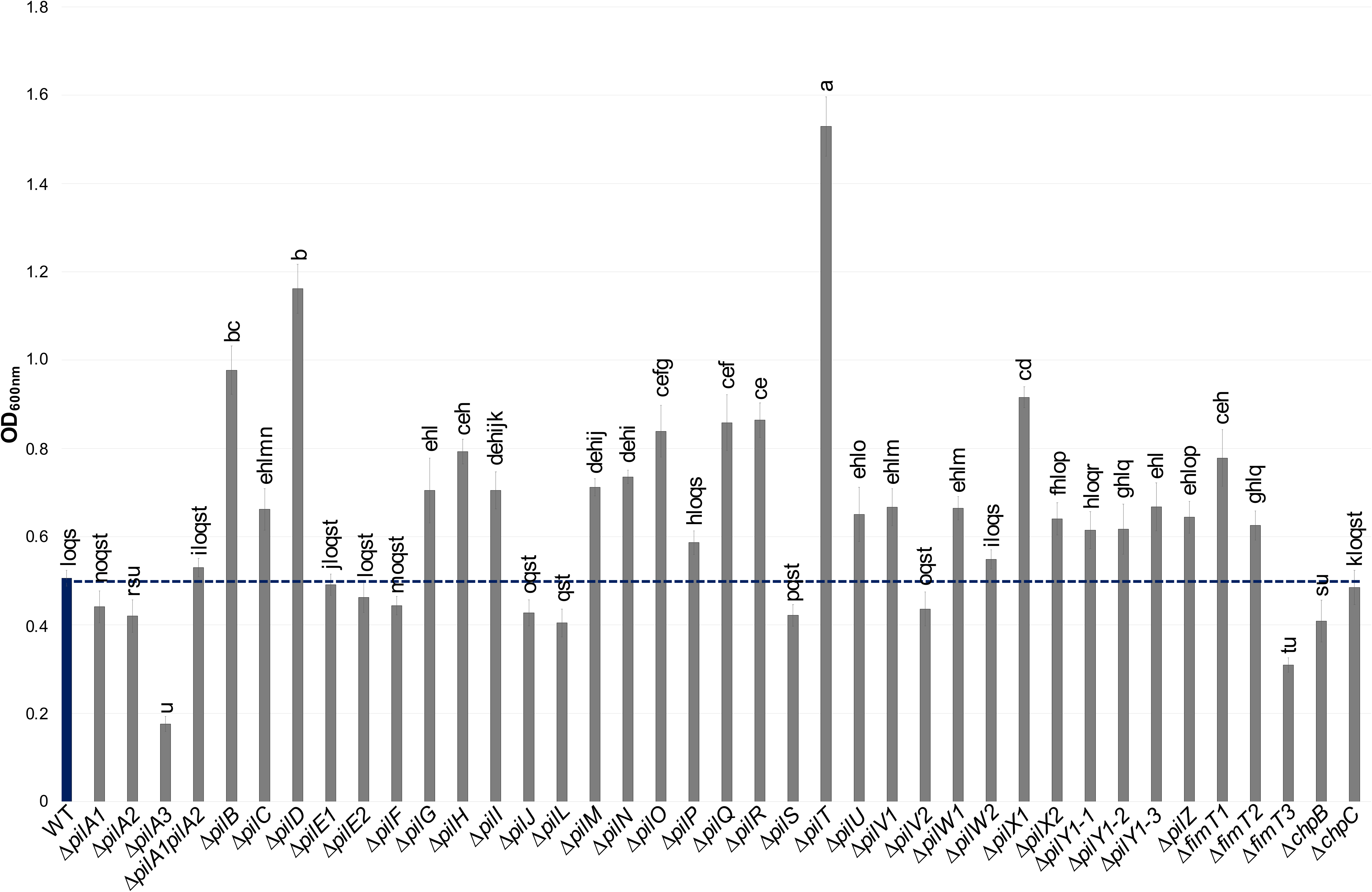

Supplement: S7 Fig — Biofilm was measured by staining X. fastidiosa cells attached to the 96-well plates at the end of the growth curve experiment with crystal violet. WT is highlighted in blue, and the dashed blue line indicates the mean value of biofilm formation for the WT. Data represent means and standard errors. Different letters on top of bars indicate significant difference as analyzed by ANOVA followed by Tukey’s HSD multiple comparisons of means (P<0.05; n = three to 14 independent replicates with six to eight internal replicates each). (TIF) [file ppat.1011154.s015.tif]

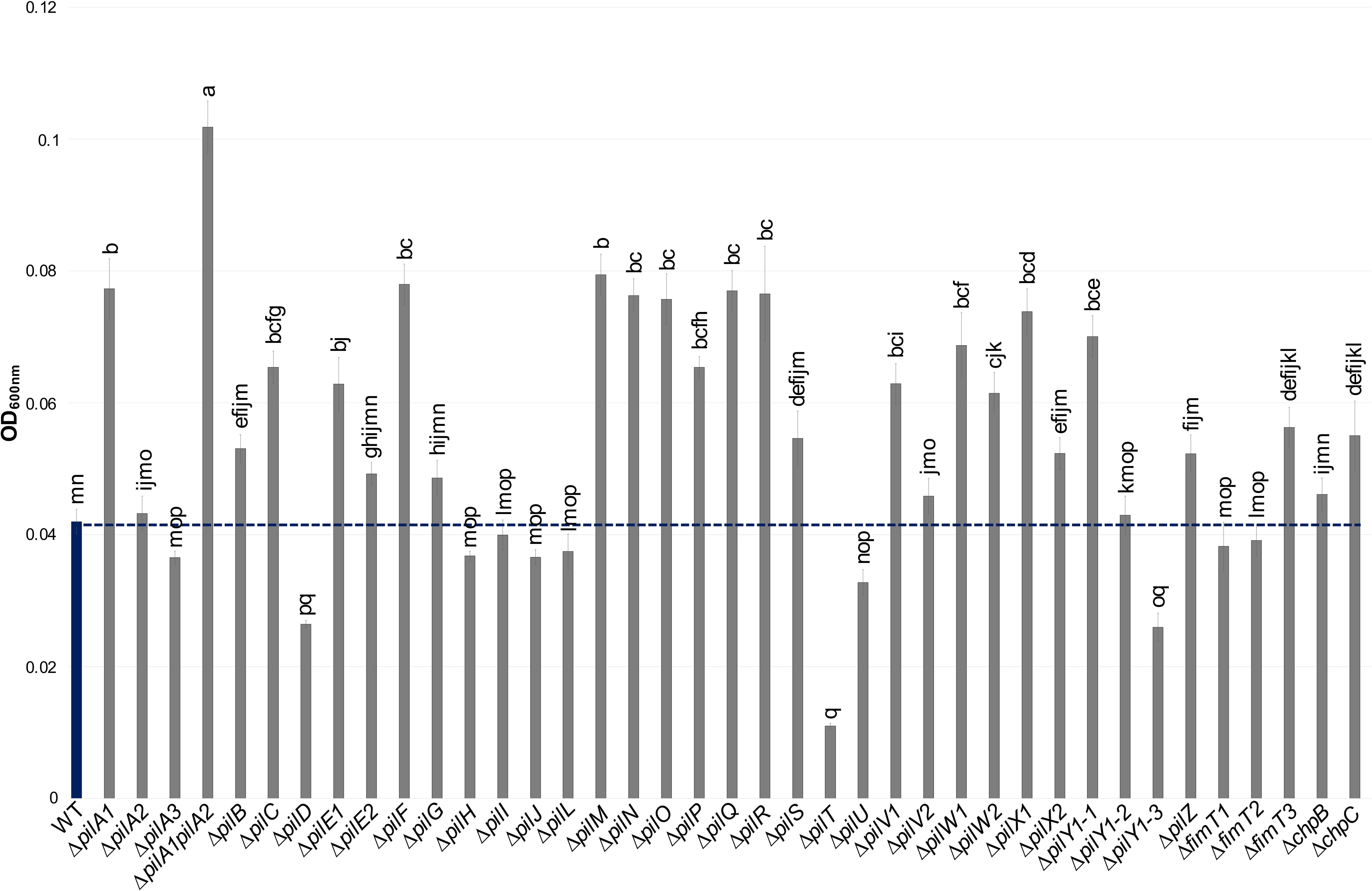

Supplement: S8 Fig — Planktonic growth was quantified by measuring the optical density at 600 nm (OD600nm) values of the supernatant of each X. fastidiosa strain at the end of the growth curve experiment. WT is highlighted in blue, and the dashed blue line indicates the mean value of planktonic growth for the WT. Data represent means and standard errors. Different letters on top of bars indicate significant difference as analyzed by ANOVA followed by Tukey’s HSD multiple comparisons of means (P<0.05; n = three to 14 independent replicates with six to eight internal replicates each). (TIF) [file ppat.1011154.s016.tif]

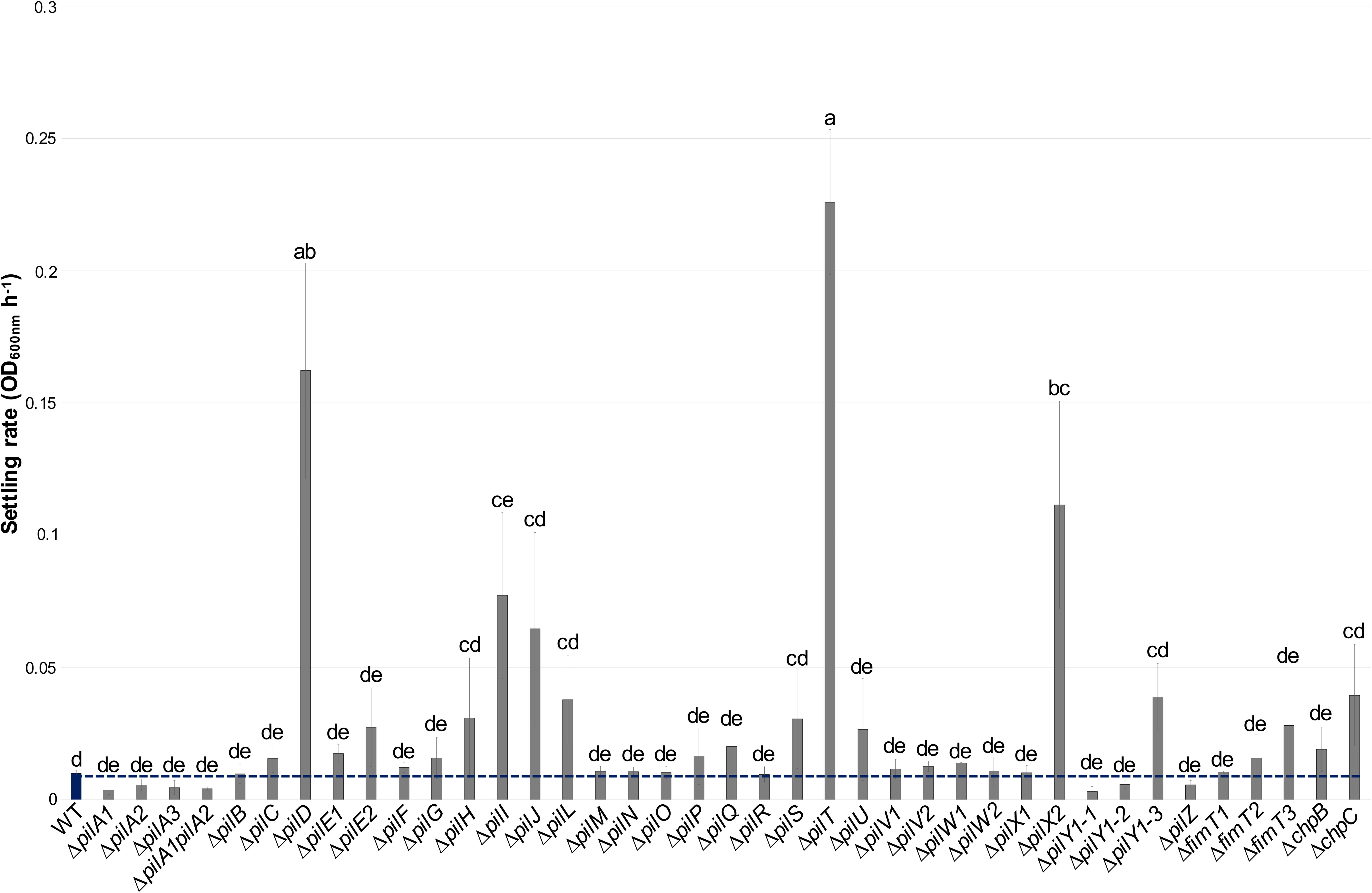

Supplement: S9 Fig — Settling rate was measured by suspending (OD600nm = 1.0) the analyzed X. fastidiosa strains in a cuvette in 1 ml of PD3 broth and measuring OD600nm values at the initial time point and after 2 hours. WT is highlighted in blue, and the dashed blue line indicates the mean value of settling rate for the WT. Data represent means and standard errors. Different letters on top of bars indicate significant difference as analyzed by ANOVA followed by Tukey’s HSD multiple comparisons of means (P<0.05; n = three to 15 independent replicates). (TIF) [file ppat.1011154.s017.tif]

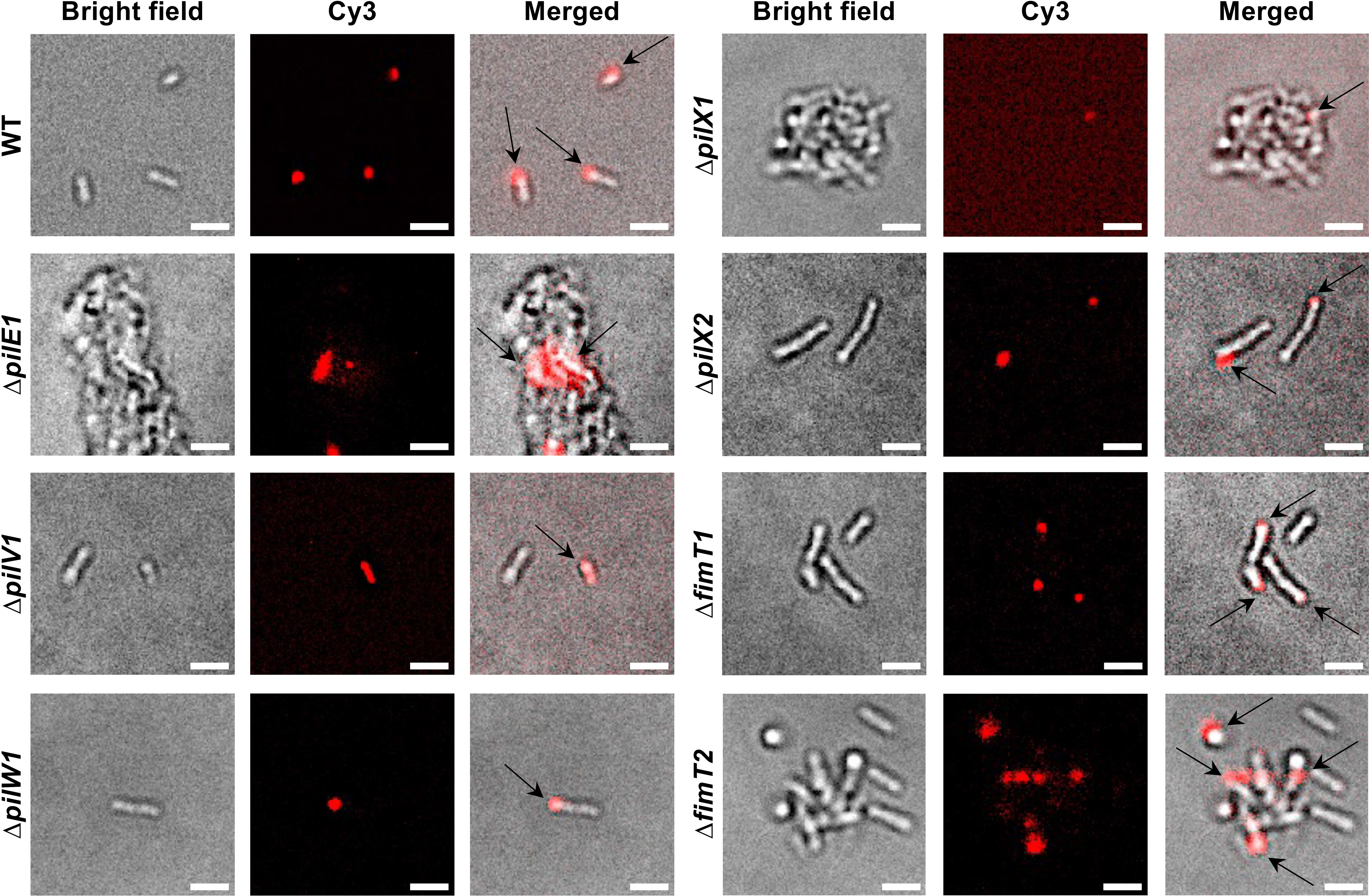

Supplement: S10 Fig — WT and mutant cells were exposed to Cy-3-labeled pAX1-Cm plasmid (1 μg) for 24 hours, treated with DNase I and DNA foci were observed using a fluorescence microscope. The images shown correspond to the bright field (left), Cy-3 channel (center) and merged fluorescent images (right). In merged fluorescent images, arrows are pointing to fluorescent DNA foci at the poles of cells. Similar events were captured in two to three independent experiments. All evaluated cells presented uptake of Cy-3 labeled DNA. Mutant strains analyzed here correspond to knockout of minor pilins that presented lower recombination through natural competence in comparison to the WT. ΔfimT1 and ΔfimT2 were included for comparison with ΔfimT3. Images were captured at 100× magnification. Scale bars, 1.5 μm. (TIF) [file ppat.1011154.s018.tif]

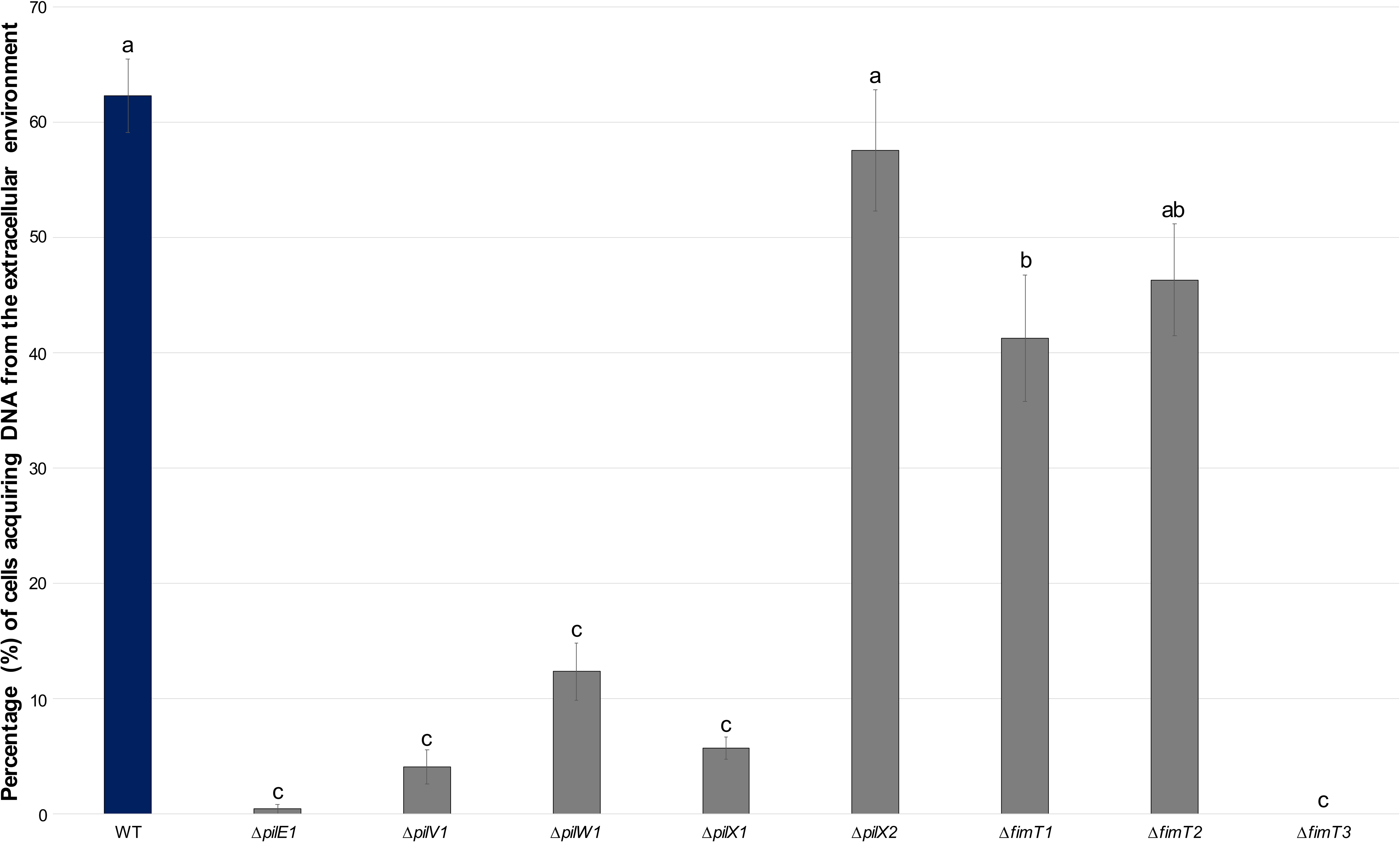

Supplement: S11 Fig — Total cells and cells with DNA foci (Cy-3 labeled pAX1-Cm plasmid) were counted and results are expressed as the percentage of cells with DNA foci. All mutants of minor pilins apart ΔpilX2 and ΔfimT2 presented significant lower DNA uptake than WT cells. Data represent means and standard errors. Different letters on top of bars indicate significant difference as analyzed by ANOVA followed by Tukey’s HSD multiple comparisons of means (P<0.05; n = two to three independent replicates with three to seven technical replicates each). (TIF) [file ppat.1011154.s019.tif]

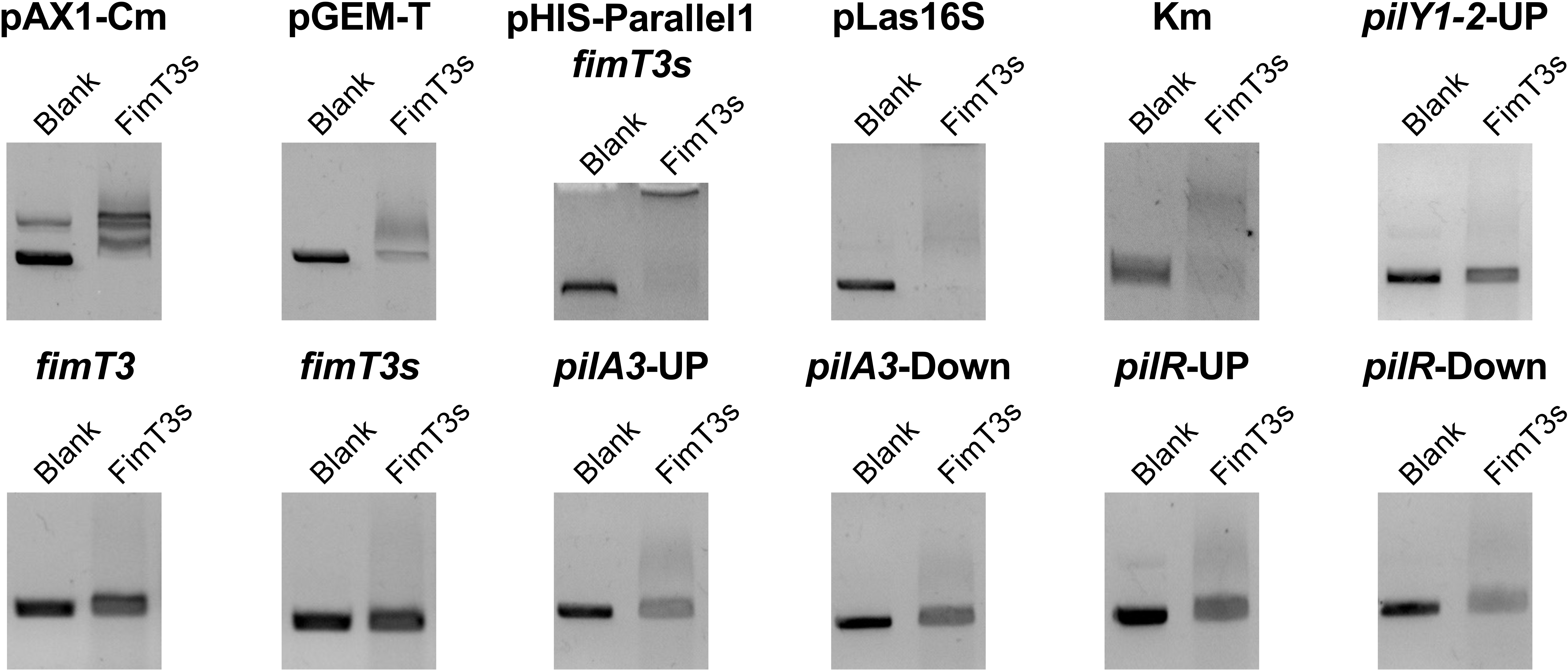

Supplement: S12 Fig — DNA-binding activity of purified FimT3s (5 μM) to different DNA sequences was assessed by agarose EMSA using standard amounts (200 ng) of each DNA sequence. FimT3s was incubated with each DNA sequence for 30 min at 28°C and resolved by electrophoresis on a 0.8% agarose gel. Lysis buffer containing 250 μM imidazole, in which proteins were suspended, was included as blank control. DNA binding activity of FimT3s in large sequences (larger than 1,200 bp) is observed as clear shifts in the electrophoretic mobility of bands, while DNA binding activity of FimT3s in small sequences (smaller than 900 bp) is observed as smearing of bands. Amplicon sizes: pAX1-Cm– 4,361 bp; pGEM-T– 3,000 bp; pHIS-Parallel1-fimT3s – 5,977 bp; pLas16S – 5,206 bp; Km– 1,202 bp; pilY1-2-UP– 988 bp; fimT3–609 bp; fimT3s – 519 bp; pilA3-UP– 852 bp; pilA3-Down– 887 bp; pilR-UP– 900 bp; pilR-Down– 937 bp. The experiment included DNA sequences with homology to the genome of X. fastidiosa TemeculaL (pAX1-Cm, pHIS-Parallel1-fimT3s, pilY1-2-UP, fimT3, fimT3s, pilA3-UP, pilA3-Down, pilR-UP and pilR-Down), as well as sequences with no apparent homology (pGEM-T, pLas16S and Km resistance cassette). FimT3s did not appear to preferably bind certain DNA sequences, indicating that its DNA-binding ability is non-sequence-specific. fimT3 –full-length fimT3 amplified from X. fastidiosa TemeculaL; fimT3s –soluble portion of fimT3 amplified from X. fastidiosa TemeculaL; sequences labeled with “UP” and “Down” correspond to upstream and downstream sequences, respectively, used to construct the targeting sequences for site-directed mutagenesis of each gene of interest. Similar events were captured in two independent experiments. (TIF) [file ppat.1011154.s020.tif]

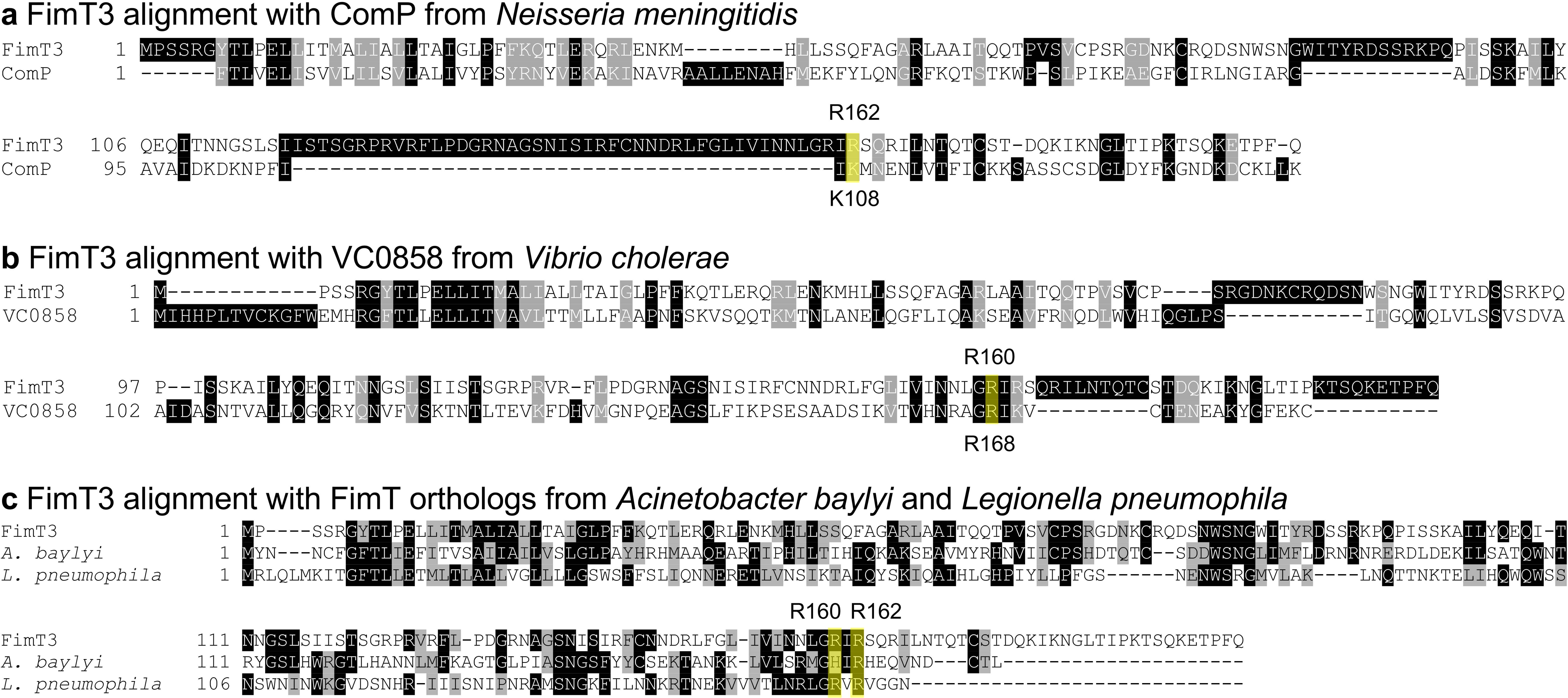

Supplement: S13 Fig — The sequence of each protein was obtained from NCBI, aligned through T-Coffee, and visualized using BoxShade. Black shading indicates conserved residues; grey shading indicates conservative mutations; and white color indicates divergence among sequences. a, b and c show alignment of FimT3 with ComP, VC0858, and FimT from A. baylyi and L. pneumophila, respectively. The arginine residue at position 162 (R162) from FimT3 aligned with a lysine of ComP (K108) demonstrated to be essential for the DNA-binding ability of the latter protein. On the other hand, the arginine residue at position 160 (R160) from FimT3 aligned with an arginine of VC0858 (R168) demonstrated to be important for the DNA-binding ability of the V. cholerae pilus. In addition, R160 aligned with an arginine residue of FimT from A. baylyi, and both R160 and R162 aligned with arginine residues of FimT from L. pneumophila that are important to its DNA-binding ability. Alignment of these specific amino acid residues are highlighted by a yellow shading in a, b and c. (TIF) [file ppat.1011154.s021.tif]

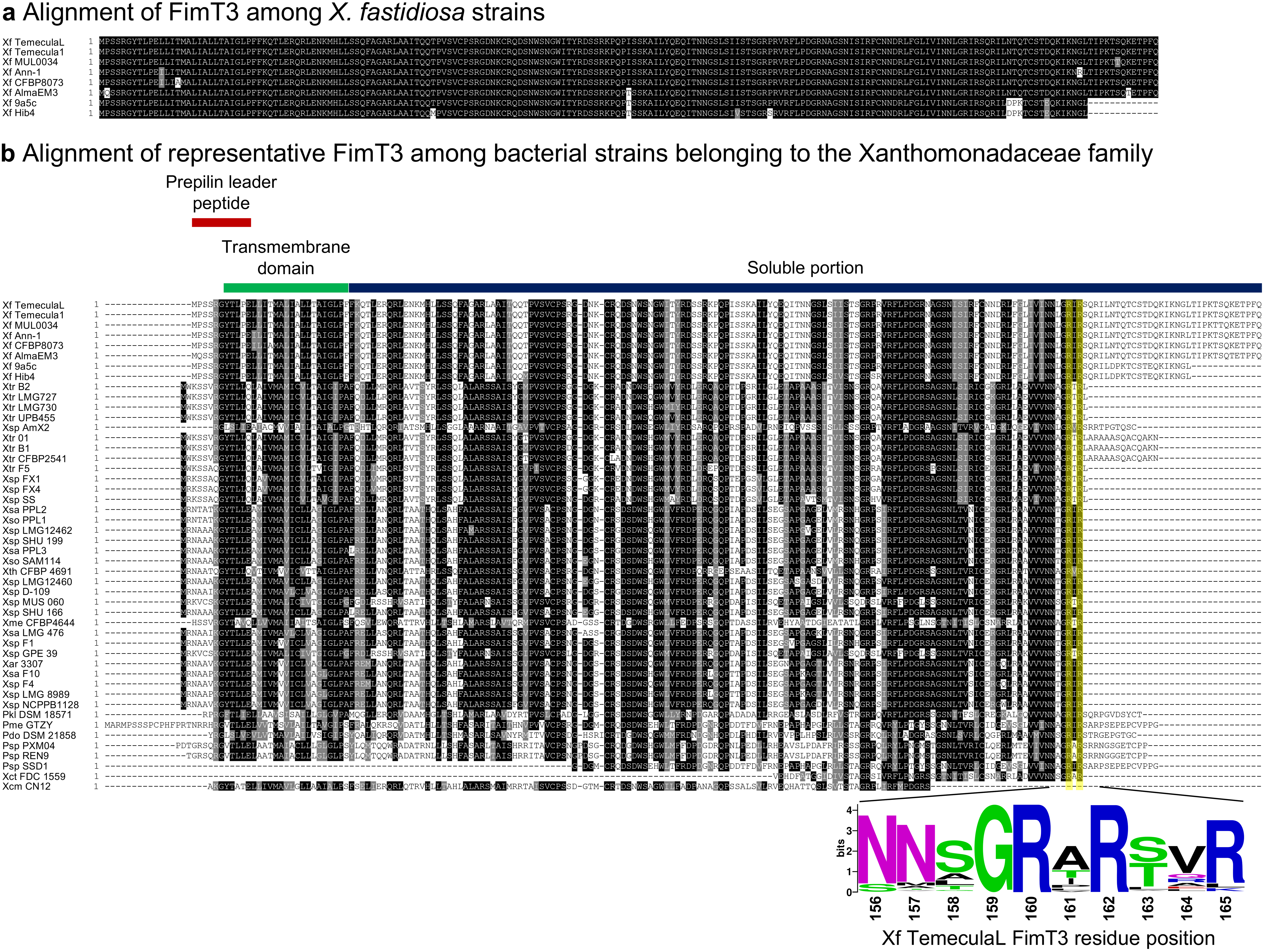

Supplement: S14 Fig — The sequence of each protein was downloaded from NCBI, screened for the presence of FimT3 by tblastn, aligned through MAFFT, and visualized using BoxShade. Black shading indicates conserved residues; grey shading indicates conservative mutations; and white color indicates divergence among sequences. a Alignment of different FimT3 sequences among X. fastidiosa strains. FimT3 is nearly identical in all X. fastidiosa strains, with strains from subspecies pauca (strains 9a5c and Hib4) presenting the highest divergence (97.25% and 95.6% of identical amino acids, respectively, in comparison to FimT3 from strain TemeculaL). b Alignment of representative FimT3 sequences from bacterial members of the Xanthomonadaceae family encoding this protein. Although the percentage of identical amino acids ranged from 15.5% to 100%, most sequences aligned with the arginine amino acid residues at positions 160 and 162 of FimT3 from X. fastidiosa strain TemeculaL (highlighted with a yellow shading in the figure). This indicates that these arginine residues are highly conserved within FimT3 sequences. For conciseness of the figure, only representative FimT3 sequences are shown in the alignment. The different portions of FimT3 (prepilin leader peptide, transmembrane domain, and soluble portion) are indicated in the figure. A sequence logo generated using the full alignment of 1,416 FimT3 sequences to highlight the conserved GRxR motif is shown in the bottom of the figure. Abbreviations are the following. Pseudoxanthomonas: Pdo–P. dokdonensis; Pkl–P. kalamensis; Pme–P. mexicana; Psp–P. sp. Xanthomonas: Xar–X. arboricola; Xcm–X. campestris; Xct–X. citri; Xme–X. melonis; Xsa–X. sacchari; Xso–X. sontii; Xsp–X. sp.; Xth–X. theicola; Xtr–X. translucens. Xylella: Xf–X. fastidiosa. (TIF) [file ppat.1011154.s022.tif]
