# Supplementary material for: Complete functional analysis of type IV pilus components of a reemergent plant pathogen reveals neofunctionalization of paralog genes
Source: PLoS Pathog. 2023 Feb 13;19(2):e1011154. doi: 10.1371/journal.ppat.1011154 (PMC9956873; doi:10.1371/journal.ppat.1011154)
Supplement: Attachment — Submitted filename: Comments.docx [file ppat.1011154.s023.docx]

The present study “Complete functional analysis of type IV pilus components of a reemergent plant pathogen reveals neofunctionalization of paralog genes” represents experiments by Marcus V, Merfa et al. This study demonstrated the roles of the 38 genes of Type IV pilus (TFP) of the Xylella fastidiosa in movement, natural competence and virulence The authors showed that ten genes were essential for both movement and natural competence, but seven sets of paralogs showed opposing phenotypes, indicating evolutionary neofunctionalization of subunits within TFP. The authors determined that the minor pilin FimT3 is the DNA receptor in TFP, and it is conserved among X. fastidiosa strains and can bind DNA in a non-specifically pattern. Two arginine residues in FimT3 are essential for DNA binding. I think the work is very important and valuable, also provided some insights in T4P function and also have referential value for other bacteria such as Xanthomonas oryzae pv. oryzae living under flow conditions. Just there are a little advice for authors some results such as Result 1 was written too complicated
