# Supplementary material for: Complete functional analysis of type IV pilus components of a reemergent plant pathogen reveals neofunctionalization of paralog genes
Source: PLoS Pathog. 2023 Feb 13;19(2):e1011154. doi: 10.1371/journal.ppat.1011154 (PMC9956873; doi:10.1371/journal.ppat.1011154)
Supplement: Attachment — Submitted filename: Response to reviewers comments - Merfa.docx [file ppat.1011154.s024.docx]

***Re:* Manuscript PPATHOGENS-D-22-01967, “Complete functional analysis of type IV pilus components of a reemergent plant pathogen reveals neofunctionalization of paralog genes”**

We would like to thank the editor and the three reviewers for their careful review and kind words and suggestions towards our work. We have made the corrections and adjustments, as suggested by the reviewers and the editor, and clarified their questions to the best of our knowledge. We believe that these changes and corrections have improved the quality of our paper, and we are very grateful for this.

**Note**: Specific comments taken from the editor and reviewers’ comments are included here in bold, followed by their respective responses. Line numbers of the corrections correspond to the Marked-up Manuscript version using track changes (“All Markup” option).

**Editor’s comments to authors:**

**Dear Dr De La Fuente,**

**Thank you very much for submitting your manuscript "Complete functional analysis of type IV pilus components of a reemergent plant pathogen reveals neofunctionalization of paralog genes" for consideration at PLOS Pathogens. As with all papers reviewed by the journal, your manuscript was reviewed by members of the editorial board and by several independent reviewers. The reviewers appreciated the attention to an important topic and were overall impressed by both the quality and presentation of the data. Based on the reviews, we are likely to accept this manuscript for publication, provided that you consider the minor suggestions of reviewers #1 and #3 and revise the manuscript accordingly.**

We thank the editor for carefully choosing expert reviewers to comment on our manuscript. Please refer below to our responses to the suggestions made by the reviewers.

**Reviewers’ comments to authors**

**Reviewer #1**

**Type IV pilus(TFP) involve in twitching motility, adhesion and even DNA exchange. The manuscript functional analyzed the TFP-relative genes in Xylella fastidiosa through detecting twitching motility, bacterial growth, virulence and also the natural transformation of a serious of mutant strains. Generally, the authors had done many experiments to identify TFP-relative gene function. And the work is interesting to reader and relative researchers.

Comment #1: In Fig 1, the authors displayed the fringe width and recombination frequency of mutant strains in a combined figure. It is much clearer and more readable for reader to divide natural transformation and motility phenotype into Fig 1A and Fig 1B.**

We thank the reviewer for the suggestion; however, the aim of this figure was to show the correlation between these two phenotypes, movement and natural transformation. Thus, we think it is important to keep the figure as is in the main body of the manuscript. Noteworthy, in our manuscript, we included separate figures for each phenotype quantification that are available in the supplemental Figures S1, S2 and S3, which were kept in this revised version of our work and are referenced throughout the manuscript.

**Comment #2: For the DNA-binding assay, why are there always a band locating at the protein size even at 0 uM protein in Fig. 2C and 2E?**

We appreciate the reviewer’s comment, that prompt us to clarify this result. We used the pAX1-Cm plasmid in our DNA-binding assays. After isolation, plasmids usually exist in three forms: supercoiled, linear, and open circular state, with each displaying bands of different sizes in agarose gels. That is the reason why we observed multiple bands in our gels even when using 0 µM protein. We have added this information to the caption of Figure 3 (previous figure 2) for clarification (ln. 768-770). Without protein added (0µM) we observe the plasmid alone. After addition of the purified FimT3 protein, we observed the change in mobility of the plasmid DNA.

**Comment #3: For statistic analysis, the author described in Line 705, 809, 818, 829, 845, 852, 859 and 867 as “n = three to ** independent replicates with two internal replicates each”. It is more incredible or reasonable to give the detail number of replicates for each experiment.**

We had different number of replicates for different strains because we had to analyze sets of mutant strains independently, with each set always containing the wild type strain for reference. Thus, each strain was analyzed at least in three independent replicates, each with a varying number of internal replicates depending on the experiment. By doing that, the wild type was always analyzed in more independent replicates than the mutant strains. Nonetheless, we made sure that every strain was analyzed independently at least three times. We believe that if we included the number of replicates for each mutant strain separately from the wild type it would be confusing and will add a considerable amount of text.

**Comment #4: Both minor pilins encoding gene pilE1 and fimT3 are required for the natural competence of acquiring DNA from extracellular environment (Fig S13), can PilE1 bind different DNA sequences similar as FimT3?**

Figure S13 (now figure S12) is demonstrating the different DNA molecules to which FimT3s was able to bind. The reviewer may be referring to figure S11 (previous figure S12), where we indeed saw a decrease (but not disruption) of DNA acquisition by the ∆*pilE1* mutant strain. This also happened with other analyzed mutant strains of minor pilins. However, in these other mutants, twitching motility was also significantly reduced or totally disrupted (figures 1, S2 and S3), which suggests an overall disruption of the type IV pilus structure and not a specific role in DNA binding itself. This is probably due to the role of minor pilins in assembling the type IV pilus structure. On the other hand, the ∆*fimT3* mutant strain is the only analyzed strain in which natural competence was abrogated without disrupting twitching motility. In fact, twitching motility was surprisingly higher in this mutant. Although we cannot discard that other minor pilin(s) could bind DNA, FimT3 was the only type IV pilus component which was essential only for natural competence, and not twitching motility, suggesting a role in DNA binding. This was described in the original manuscript in ln. 145-147 and ln. 211-214. The DNA binding role was also supported by an in silico analysis (ln. 250-252 of the original manuscript), which did not support DNA binding by any other minor pilin of *Xylella fastidiosa*. Therefore, we do not think that PilE1 can also bind DNA.

**Comment #5: In Fig S10C and S10D, no significant difference of bacterial population of WT and mutant strains was found in top leaf. However, the symptoms caused by inoculation with different mutant strains displayed the clear differences.**

Yes, that is correct. We think that the difference between strains populations and symptoms is due to the experimental design. We have previously condensed this information in ln. 205-208 of the revised manuscript. We have also included a discussion of these results in our supplementary information text (quoted below).

“The functional role of some TFP genes in the virulence in planta of *X. fastidiosa* has also been previously investigated. Deletion of *pilG* and *pilL*, which abrogated twitching motility in previous studies ^36,44^, resulted in avirulence and delayed, less severe symptoms in infected grapevines, respectively ^36,67^. Moreover, the non-motile ∆*pilB* and ∆*pilQ* mutants have significantly reduced basipetal translocation in planta, which occurs in a reverse direction away from the leaves and thus against the flow of xylem sap ^68^. Similarly, ∆*pilA*, ∆*pilQ* and ∆*pilT* mutants in *R. solanacearum*, which are all impaired in twitching motility, also presented reduced virulence in tomato plants ^69,70^. However, all these studies were performed with twitching motility-deficient mutant strains. Here, we have assayed the virulence of *X. fastidiosa* using mutant strains with variable phenotypes, including higher movement, lower movement, and non-motility. Regardless, all presented reduced virulence in planta, except for the ∆*pilA2* mutant, which promoted similar disease severity to WT-inoculated plants. Although the results obtained for movement-deficient strains were in accordance with their twitching motility phenotypes, we expected that mutant strains with higher twitching motility would be hypervirulent. Likewise, *X. fastidiosa* mutant strains lacking the *rpfF* gene, which encodes the diffusible signal factor (DSF; quorum sensing molecule of this bacterium) ^71^ synthase, have increased motility and are hypervirulent in planta ^57,71-73^. DSF negatively regulates twitching motility in *X. fastidiosa* and positively modulates biofilm formation ^71^. Thus, the higher virulence of ∆*rpfF* is linked to increased movement of *X. fastidiosa* within infected plants and higher colonization of xylem vessels ^72,73^. In our study, however, we have mutated genes directly linked to TFP assembly and functioning, and not genes upstream of these processes. Also, we did not observe significant impairment in the colonization of infected plants by the analyzed mutant strains. This may be due to experimental bias, since we have inoculated basal leaves of young tobacco plants, which possibly allowed *X. fastidiosa* cells to colonize the plants by moving with the flow of xylem sap. Therefore, it would be interesting to complement our study in the future by inoculating the canopy of full-grown plants and observing whether colonization will be impaired or enhanced due to the observed changes in twitching motility in vitro upon deletion of TFP-related genes, which may be reflected in their basipetal movement. Together, the fact that twitching motility-deficient strains, as well as mutant strains with higher movement, had reduced virulence in this study indicate that TFP genes are required for the proper functioning and regulation of this machinery, which is needed for full symptom development; whilst deletion of its individual components suppresses virulence”.

**Reviewer #2**

**This study demonstrated the roles of the 38 genes of Type IV pilus (TFP) of the Xylella fastidiosa in movement, natural competence and virulence The authors showed that ten genes were essential for both movement and natural competence, but seven sets of paralogs showed opposing phenotypes, indicating evolutionary neofunctionalization of subunits within TFP. The authors determined that the minor pilin FimT3 is the DNA receptor in TFP, and it is conserved among X. fastidiosa strains and can bind DNA in a non-specifically pattern. Two arginine residues in FimT3 are essential for DNA binding. I think the work is very important and valuable, also provided some insights in T4P function and also have referential value for other bacteria such as Xanthomonas oryzae pv. oryzae living under flow conditions.**

**Comment #1: Just there are a little advice for authors some results such as Result 1 was written too complicated.**

We thank the reviewer for the suggestion. We hope that the edits we performed to the manuscript made it clearer for readers.

**Reviewer #3**

**This study has reported on the results of a rather comprehensive study of the various roles of type IV pili (TFP) in the important plant pathogenic bacterium Xylella fastidiosa. This study follows extensive prior work by this group and by others that have zeroed in on the important role of TFP in this pathogen both because of the important roles of TFP in attachment to surfaces, and in active movement of the pathogen in xylem vessels subject to high velocity liquid flow. This group also has contributed nearly everything that we know about the role of competency in this species, which complements the extensive genomic sequencing studies that have revealed a particularly large role of recombination and horizontal gene transfer in this species. This current study therefore is highly justified as it would be expected that TFP would play a role both in active motility and DNA uptake. What makes a study particularly impressive is the comprehensive nature by which the authors have identified and individually knocked out each of the many genes involved in TFP production and made functional assessments of the mutants. The study is particularly impressive because of the quantitative nature of their measurements of the contribution of each of these genes to both competency, as measured by recombination frequency and active motility, measured by quantitative twitching assays. The data presented in Figure 1 is this a real tour de force. While I found this aspect of this study to be the most biologically interesting and compelling, the authors also have presented reasonable data to support the conclusions that the minor pilus FimT3 is THE DNA receptor in TFP of X. fastidiosa. While I don't think there is any real argument that FimT3 is in fact the DNA receptor, I felt that they were straining a bit too try to demonstrate the essentiality of the two arginines in locations 160 and 162 in the data presented in lines 255 to 270. They seem to agree that the data and support of these two residues are somewhat soft, as represented in the very modest defects of alanine substitutions on gel shift studies shown in Figure 2. While the data is rather soft because of the very small effect of these substitutions, I don't have any argument in retaining it in the study. Perhaps I am simply not as interested in the phylogenetic components of the study, but the data addressing the divergent and conservation of fimT3 in X. fastidiosa, other Xanthomonadacea, and more broadly in bacteria didn't seem to really add too much to the story, although it did seem to explain why subspecies pauca is not competent. The arguments for neofunctionalization of these various TFP components is compelling. Their study does provide fodder for some interesting follow-on studies. Specifically, their finding that the presence of paralogs for major and minor pilins having opposite functions seem to suggest that there could be assembly of multiple TFP fibers having specific functions. I note that the manuscript is exceptionally well written. This is a refreshing change from the many manuscripts that I get to review that are very difficult to read.**

We thank the reviewer for the kind words towards our study, and we really appreciate the understanding of the amount of work done, and the compliments on the writing of the manuscript. We agree that the arginine replacements we have performed in our study did not demonstrate their essentiality for DNA binding in *X. fastidiosa*, and we are glad that the reviewer understood our discussion of the results, and that they suggested to keep the data.

**Comment #1: Lines 49 through 50. The sentence seems to accurately summarize the results of this study, but if they have the space, I think it would have more impact if there was some a statement about whether the presence of fimT3 was linked to competency in any of these other taxa.**

This is a good suggestion. Unfortunately, the mentioned other members of the Xanthomonadaceae family have not yet been characterized for natural competence. We have previously included this information in ln. 368-369 of the original manuscript, and we have now added this information to our abstract (ln. 51-52), as suggested.

**Comment #2: Line 141. The sentence is a bit awkward in that they should indicate that deletion of pilR showed that PilR was essential for twitching mobility but not transformation.**

We have edited this sentence for clarification (ln. 143).

**Comment #3: Lines 173 through 175. The authors have not provided enough information in the materials and methods for me to evaluate whether they have indeed overcome the complications of the likely clumping of cells in these assays. X. fastidiosa is notorious for cell-cell adhesion. The authors clearly understand that growth rate measurements done by measuring optical density is prone to issues, but it is not clear how their simple measurement of CFU/milliliter by dilution plating overcomes this issue unless there was an attempt to disperse the cells by sonication or some other method to disassociate the cells. CFU/ml can differ hugely from cells/ml, which is their goal here, if in fact the cells have not been dispersed.**

We agree with the reviewer’s comment, and to be clear, we do not think we overcame the problems with clumping of *X. fastidiosa* cells. We just wanted to show two different methodologies used for cell quantification. To minimize the cell-cell adhesion effects in our assays when preparing bacterial suspensions and performing serial dilution plating, cells were always resuspended by thorough pipetting followed by vortexing to disperse cells and foster greater homogenization. This information has been added to the Materials and Methods section in ln. 401-403. We did not perform sonication since we were concerned that it may be too deleterious for *X. fastidiosa* cells.

**Comment #4: Lines 189 through 206. I was disappointed and surprised that they did not place their data on the virulence measurements of these strains in a figure in the text rather than as a supplemental figure. I think a lot of readers would like to see this type of information, and many would be reluctant to dig into the supplement to find it. Since they only have a small number of figures already, I think it would be better to put it in the main text.**

We thank the reviewer for the suggestion and have moved the virulence data out of supplementary materials into the main manuscript (current Fig. 2).

**Comment #5: Lines 215-217. The sentence is awkward. It would seem that they have used DNase1 to remove any extracellular DNA, but this sentence does not make that clear.**

That is correct. We have edited this sentence for clarification (ln. 217-218).

**Comment #6: Line 227. ... and compare it to its paralogs..**

Done (ln 229).

**Comment #7: Line 280 ... of the Xanthomonadaceae ( 47%), the majority...**

Done (ln. 283).
